# Active Faulting and Deep‐Seated Gravitational Slope Deformation in Carbonate Rocks (Central Apennines, Italy): A New “Close‐Up” View

**DOI:** 10.1029/2021TC006698

**Published:** 2021-10-05

**Authors:** Luca Del Rio, Marco Moro, Michele Fondriest, Michele Saroli, Stefano Gori, Emanuela Falcucci, Andrea Cavallo, Fawzi Doumaz, Giulio Di Toro

**Affiliations:** ^1^ Dipartimento di Geoscienze Università degli Studi di Padova Padua Italy; ^2^ Istituto Nazionale di Geofisica e Vulcanologia (INGV) Rome Italy; ^3^ Institut des Sciences de la Terre (ISTerre) Universitè Grenoble Alpes Grenoble France; ^4^ Università degli Studi di Cassino Cassino Italy; ^5^ Laboratorio tecnologico multidisciplinare CERTEMA Grosseto Italy

**Keywords:** active faults, deep‐seated gravitational slope deformation, earthquakes, slip surfaces, cataclasites, carbonates

## Abstract

Active faulting and deep‐seated gravitational slope deformation (DGSD) are common geological hazards in mountain belts worldwide. In the Italian central Apennines, kilometer‐thick carbonate sedimentary sequences are cut by major active normal faults that shape the landscape, generating intermontane basins. Geomorphological observations suggest that the DGSDs are commonly located in fault footwalls. We selected five mountain slopes affected by DGSD and exposing the footwall of active seismogenic normal faults exhumed from 2 to 0.5 km depth. Field structural analysis of the slopes shows that DGSDs exploit preexisting surfaces formed both at depth and near the ground surface by tectonic faulting and, locally, by gravitational collapse. Furthermore, the exposure of sharp scarps along mountain slopes in the central Apennines can be enhanced either by surface seismic rupturing or gravitational movements (e.g., DGSD) or by a combination of the two. At the microscale, DGSDs accommodate deformation mechanisms similar to those associated with tectonic faulting. The widespread compaction of micro‐grains (e.g., clast indentation), observed in the matrix of both normal faults and DGSD slip zones, is consistent with clast fragmentation, fluid‐infiltration, and congruent pressure‐solution active at low ambient temperatures (<60°C) and lithostatic pressures (<80 MPa). Although clast comminution is more intense in the slip zones of normal faults because of the larger displacement accommodated, we are not able to find microstructural markers that allow us to uniquely distinguish faults from DGSDs.

## Key Points

- Slip surfaces cutting carbonate rocks in Central Italian Apennines accommodate both normal faulting and deep‐seated gravitational slope deformations (DGSDs)
- DGSDs are the result of gravity‐induced reactivation of faults and fractures located in the footwall of normal seismogenic faults
- Deformation mechanisms associated with normal faulting and DGSDs are cataclasis and pressure‐solution active at low ambient temperatures

## 1 Introduction

Deep‐seated gravitational slope deformations (DGSDs) are large and slow rock‐mass movements (slip rate of a few mm/year) commonly affecting the entire relief slope and involving ∼200–300 m thick rock volumes, with relatively small displacements compared to the slope dimensions (Agliardi et al., 2001, 2012; Dramis & Sorriso‐Valvo, 1994; Varnes, 1978; Zischinsky, 1966, 1969). Unlike other types of landslides, DGSDs commonly lack clearly defined boundaries (Crosta et al., 2013). Sackung‐type DGSDs are commonly produced by slow rock‐mass movements that occur on high and steep slopes from the ridge crest to the valley floor and result in the bulging of the lower sector of the slope (Agliardi et al., 2012; Hermann et al., 2000; Savage & Varnes, 1987; Zischinsky, 1969). On the other hand, lateral spreading DGSDs are due to lateral mass movements in areas where a thick‐bedded and gently dipping sedimentary succession overlies a less competent unit (Agliardi et al., 2012; Jahn, 1964; Zischinsky, 1969). Peculiar morphologies associated with DGSDs are double‐crested ridges, ridge‐top grabens, scarps and counterslope scarps, ridge‐parallel trenches, tension cracks, and bulging slopes (Agliardi et al., 2001, 2012). Several natural factors, and their interaction, control the formation of these large slope instabilities, such as the lithostratigraphic and structural setting (Agliardi et al., 2001; Hermann et al., 2000; Mariani & Zerboni, 2020), the topographic relief and the state of the stress (Ambrosi & Crosta, 2006, 2011; Martel, 2006; Molnar, 2004), weather and climate (Agliardi et al., 2001; Evans & Clague, 1994), and seismic faulting (Jibson et al., 2004; McCalpin, 1999). Significant natural hazards are associated with DGSDs (Ambrosi & Crosta, 2006; Dramis & Sorriso‐Valvo, 1994), in particular due to a sudden acceleration of the slope movements commonly induced by seismic faulting (Chigira et al., 2010; Moro et al., 2007). A large number of DGSDs were documented worldwide since 1990s, in particular in North America, Europe, Japan, and New Zealand (see Figure 1 from Panek & Klimeš, 2016). Italy is one of the countries in which DGSDs have been commonly monitored and reported, both in the Alps (Crosta et al., 2013; Mariotto & Tibaldi, 2015) and in the Apennines (Aringoli et al., 2010; Bianchi Fasani et al., 2014; Della Seta et al., 2017; Esposito et al., 2007; Galadini, 2006; Mariani & Zerboni, 2020; Moro et al., 2007, 2009, 2012; Gori et al., 2014).

**Figure 1:**
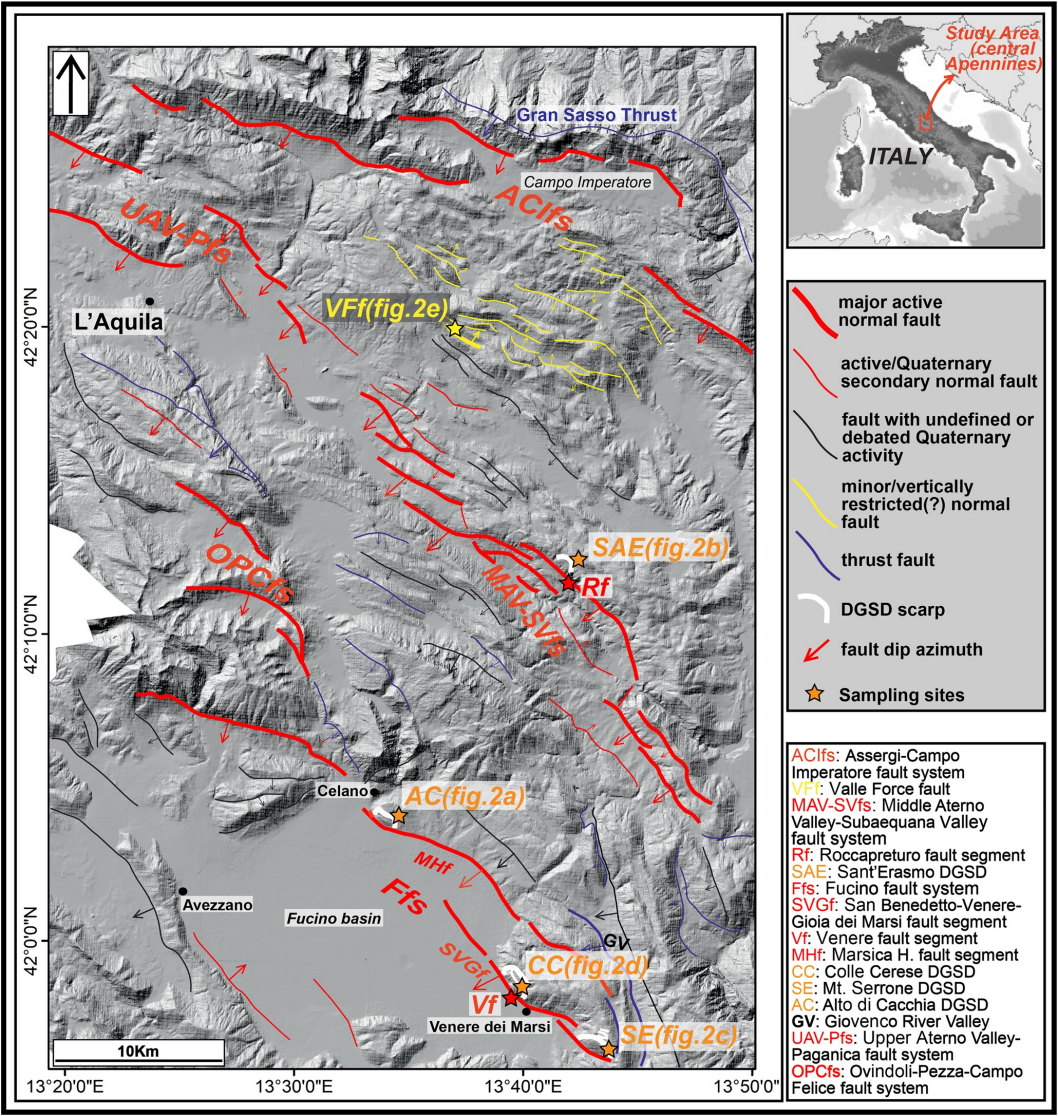
Structural scheme of the study area. Major active normal faults (thick red lines): Assergi‐Campo Imperatore fault system (Galli et al., 2008); Middle Aterno Valley‐Subaequana Valley fault system (Falcucci et al., 2015); Fucino fault system (Galadini & Galli, 1999); Upper Aterno Valley‐Paganica fault system (Moro et al., 2013); and Ovindoli‐Pezza‐Campo Felice fault system (Pantosti et al., 1996). Minor vertically restricted normal faults (black in color) are from D'Agostino et al. (1998) and Falcucci et al. (2015). Deep‐seated gravitational slope deformations (DGSDs) are widespread in this region, as well as in all the Apennines (see main text for the references). DGSDs analyzed in this work are shown in this figure. Alto di Cacchia, Sant'Erasmo, Colle Cerese, and Mt. Serrone DGSDs (White in color) are all located in the footwall of major active normal faults; Valle Force fault (yellow in color) is ∼2 km long along‐strike. Stars indicate the sampling sites of the studied slip zones (next figures).

In the central Apennines, the main controlling factor for the formation and triggering of DGSDs is the energy relief (i.e., the difference in altitude between the highest and lowest portion) of the hillslope produced by the large number of active, often seismogenic, normal faults accommodating regional extension (Galadini, 2006; Moro et al., 2007; Figure 1), coupled with strong Quaternary regional uplift (more than 1,000 m; D'Agostino et al., 2001) and with the interglacial‐glacial climate changes (Giraudi, 2001). Active normal faulting has affected the Apennines since the Late Pliocene (e.g., Barchi et al., 2000; Boncio et al., 2004; Elter et al., 1975; Galadini et al., 2000; Galli et al., 2008; Valensise & Pantosti, 2001). The Quaternary activity of normal faults is documented by the displacement of fluvio‐lacustrine deposits filling intermontane basins (Galadini, 1999) and by historical earthquakes that affected the area (e.g., 1703 *M*_w_ 6.8 L'Aquila earthquake; 1915 *M*_w_ 7.1 Avezzano earthquake; 2009 *M*_w_ 6.1 L'Aquila earthquake; Rovida et al., 2020). In the last 25 years, paleo‐seismological and geophysical analyses focused on the mapping and assessment of the seismic hazard associated with these active faults. These interdisciplinary studies also yielded information about the length, Quaternary throw, slip rate, and earthquake recurrence intervals of the faults (e.g., Barchi et al., 2000; Calamita et al., 2000; Falcucci et al., 2016; Galadini & Galli, 2000; Galadini et al., 2003; Morewood & Roberts, 2000; Pizzi et al., 2002; Roberts & Michetti, 2004). Field structural investigations of the major (up to 15–20 km long) exhumed seismogenic fault surfaces cutting carbonate rocks documented, in several cases, the presence of a belt of up to hundred meter‐thick damage zones bounding meter‐thick fault cores (Caine & Forster, 1999) accommodating most of the cumulative displacement (Agosta & Aydin, 2006; Ferraro et al., 2019; Fondriest et al., 2020) and containing multiple cm‐ to mm‐thick principal slip zones (PSZs) cut by sharp (where karstified) or polished to “mirror‐like” (where fresh) slip surfaces (Fondriest et al., 2013, 2015, 2017; Siman‐Tov et al., 2013).

Recent paleo‐seismological, geological, and geomorphological observations pointed out that some outcropping sharp scarps cutting the central Apennines carbonate rocks, commonly interpreted as surface expression of seismic faulting, can also accommodate DGSDs (Gori et al., 2014; Moro et al., 2009, 2012). Indeed, photogeological and field analyses allowed for the identification of several geomorphological features commonly associated with a DGSD (i.e., double crest lines, scarps and counterslope scarps, slope‐parallel trenches, and open fractures). Such morphological features are the expression of near‐surface deformation at the hillslope scale (e.g., their spacing is of the order of tens of meters) and are not expected to be produced by listric normal faults. In fact, some minor and major normal faults in the central Apennines are inferred to bend from dip angles of 45–70° at the surface to sub‐horizontal at 2–10 km depth (Barchi et al., 2000; Valoroso et al., 2014). As a consequence, the deep‐seated deformation accommodated by listric faults is manifested at a larger wavelength (e.g., the spacing between intra‐mountain basins is of the order of hundreds of meters to kilometers, D'Agostino et al., 1998) than the meso‐structures associated with DGSDs. Furthermore, most of the slip surfaces of the analyzed DGSDs in central Apennines affect the upper portion of the hillslope and are less than 1 km long along‐strike. Their trenches and scarps commonly show an arcuate shape at the tips and are not geometrically linked to other structures, thus indicating a possible lateral confinement of the DGSD body (Moro et al., 2009). Instead, seismogenic normal faults in central Apennines are up to 10 km long along‐strike (Barchi et al., 2000; Boncio et al., 2004; Falcucci et al., 2015), commonly arranged in *en‐échelon* patterns, and do not show evidence of lateral confinement.

In the central Apennines, the slip surfaces associated with DGSDs and faults, respectively, should be exhumed from different depths (0 to a few 100 m for DGSDs, 0–3 km for active faults; Agosta & Kirschner, 2003), and active over a different range of (a) temperatures (<15°C for DGSDs, 0–60°C for faults assuming a geothermal gradient of ∼20°C/km, typical for the central Apennines; Mancinelli et al., 2019), (b) lithostatic pressure (<15 MPa for DGSDs, 0–80 MPa for faults), and (c) slip rates (usually <mm/s for DGSDs, up to ∼1 m/s for seismic faults). Such large differences in loading conditions should result in the formation of distinctive secondary fault/fracture networks, possibly recognizable at the outcrop scale, and microstructures of the slip zones.

**Figure 2:**
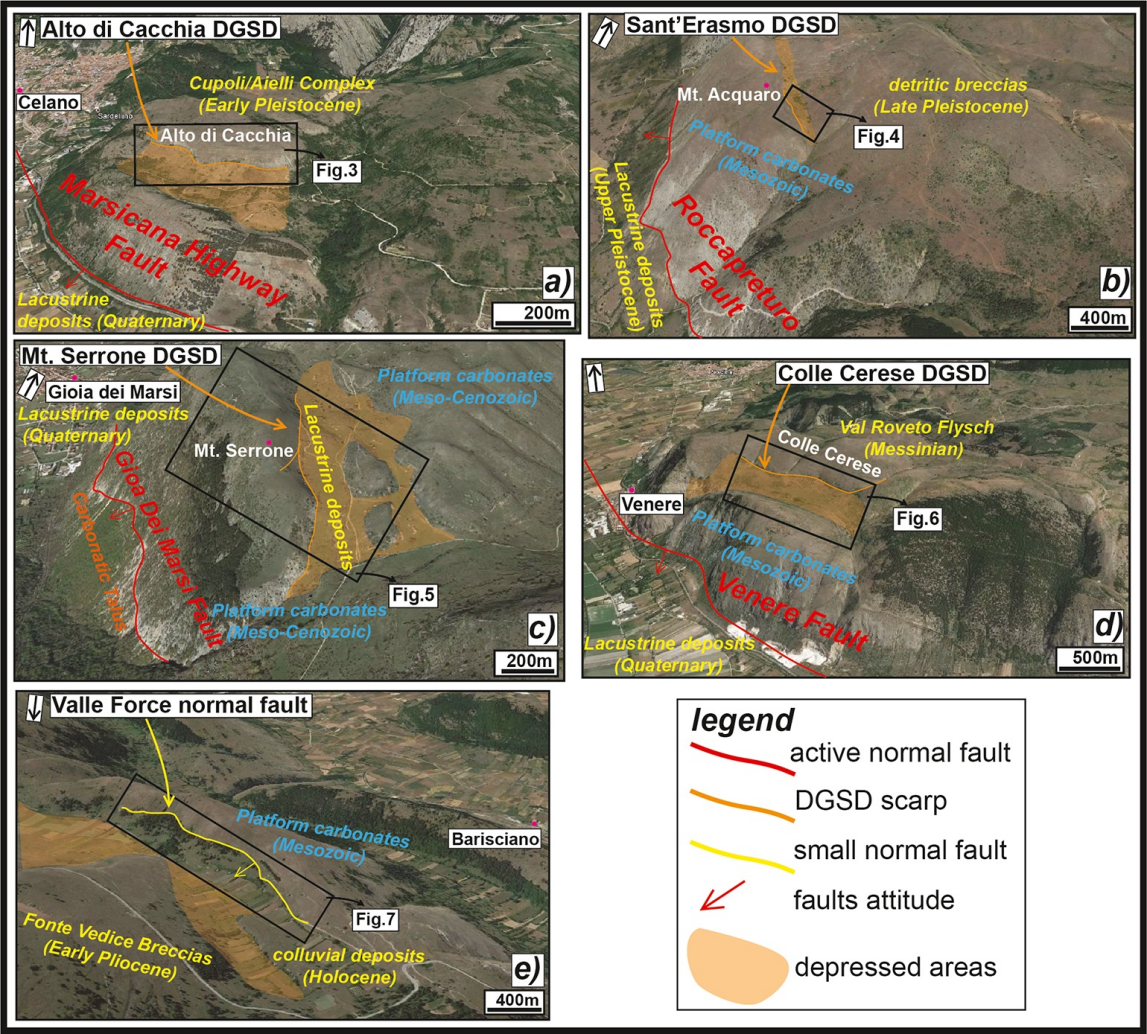
Panoramic view of the five case studies: (a) Alto di Cacchia deep‐seated gravitational slope deformation (DGSD), located in the footwall of the “Marsicana Highway” normal fault segment; (b) Sant'Erasmo DGSD, located in the footwall of Roccapreturo normal fault segment; (c) Mt. Serrone DGSD, located in the footwall of San Benedetto‐Gioia dei Marsi normal fault segment; (d) Colle Cerese DGSD, located in the footwall of San Benedetto‐Gioia dei Marsi normal fault segment (Venere sector); and (e) Valle Force normal fault, that borders a small and laterally confined basin. The lateral continuity of the DGSDs is limited compared to the associated faults. Images from Google Earth.

In this paper, we discuss four cases of DGSDs located in the footwall of active seismogenic normal faults and one case of a normal fault bordering a relatively small intermontane basin (Italian central Apennines, Figures 1 and 2). We analyzed the fracture network in the footwall of the major slip surfaces and compared the microstructures of the slip zones of the DGSDs with the ones of the associated seismic normal faults. Based on the evidence reported in our study, mesoscale structural data and microstructural analyses alone do not allow us to distinguish between structures associated with normal faults or DGSDs. Indeed, the ambient conditions at which DGSDs and near‐surface tectonic faulting occur are partially overlapping. However, the analysis of meso‐ to microstructural data set may allow us to (a) interpret how DGSD commonly form in central Apennines and (b) identify the deformation mechanisms associated with the evolution of DGSDs in space and time.

## 2 Geological Setting

The structural setting of the central Apennines is the result of the superposition of three main tectonic phases. From the Late Triassic to the Middle Jurassic, an extensional phase affecting the whole Central‐Mediterranean area related to the opening of the Liguria‐Piedmont Ocean (Western Tethys) led to the fragmentation of the Adriatic plate paleo‐margin. The diffuse normal faulting brought to drowning of some sectors of the carbonate platforms and to the formation of basins (e.g., Carminati & Doglioni, 2012; Castellarin et al., 1978; Cosentino et al., 2010). In the Late Miocene, the platform areas were involved in the Apennine orogenesis, caused by the NE‐verging convergence between the Adriatic and European plates (Carminati et al., 2012). The study area represents a platform carbonate sector of the Adriatic plate paleo‐margin that was involved in the Apennine orogenesis. The slab‐rollback of the subducting Adriatic plate caused the back‐arc opening of the Tyrrhenian Sea and the migration toward E‐NE of the Apennine fold‐and‐thrust belt (Carminati et al., 2012; Cipollari et al., 1999; Doglioni, 1995; Malinverno & Ryan, 1986; Vezzani & Ghisetti, 1998). From the Pliocene until present days, extensional tectonics accommodated the migration of the chain toward E‐NE. In the central Apennines, this extensional phase started in the Middle Pleistocene. The extension is accommodated by normal faults that cut and locally exploit the inherited Miocene‐Early Pleistocene thrusts and the earlier Mesozoic normal faults (Elter et al., 1975; Vezzani et al., 2010). The extensional Plio‐Quaternary activity was responsible for the formation of numerous intermontane basins, filled by lacustrine and alluvial deposits and bordered by large normal faults (Bosi et al., 2003; Cavinato et al., 2002; Figure 1).

The main active faults strike NW‐SE and dip toward SW, consistent with the NE‐SW direction of extension (>3 mm/year of extension rate) documented by geodetic data (D'Agostino et al., 2011; Serpelloni et al., 2005), focal mechanisms (Chiaraluce et al., 2003), and borehole breakout data (Mariucci & Muller, 2003). These normal faults cut the pre‐Miocene carbonate sequences (>4 km of stratigraphic thickness in some areas, excluding thickening due to thrust activity, Tozer et al., 2002) and caused destructive earthquakes up to moment magnitude *M*_w_ 7.1 (e.g., Avezzano 1915; Figure 1).

### 2.1 Stratigraphic and Geomorphological Characterization of the Study Cases

The four DGSDs discussed in this work were selected because they include several morphological features commonly associated with deep‐seated landslides and their major scarps strike with the same orientation of the large seismogenic faults they are associated with. Moro et al. (2009, 2012) identified double‐crested lines, scarps and counterslope scarps, slope‐parallel trenches, and open fractures associated with Colle Cerese and Mt. Serrone DGSDs, by means of Remote sensing, photogeological, and geomorphological interpretations. The four DGSDs are described below based on their stratigraphic, geomorphic, and geological setting; after that, a description of Valle Force normal fault is used for comparison.

#### 2.1.1 Alto di Cacchia DGSD

“Alto di Cacchia” is a flat top hill (950 m a.s.l.) located a few kilometer east of Celano town. The hill is about 500 m long along the NW‐SE direction and is laterally confined to WNW by the Fucino Basin and to ESE by a fluvial incision. Alto di Cacchia hill is bordered to SW by the NW‐SE oriented “Marsicana Highway” fault segment (MHf) (Galadini & Galli, 1999). The MHf, a segment of the Fucino seismogenic fault system, juxtaposes Cretaceous platform carbonates with Quaternary lacustrine deposits filling the Fucino Basin in the hangingwall (Figure 2a). The DGSD affects the uppermost portion of the Cupoli unit (∼120 m of maximum thickness), consisting of Early Pleistocene gravel intercalated with sands and silt that were deposited in lacustrine and fluvial environments (Figure 2a). The Cupoli unit both overlies and is in heteropic contact with the Aielli Conglomerates (∼400 m thick and made of blocks of carbonate rocks within a silty and clayey matrix: Bosi & Messina, 1991; Bosi et al., 2003). The geomorphological structure of the Alto di Cacchia hillslope was interpreted by us as DGSD based on the following evidences: (a) the continental deposits of the Alto di Cacchia are cut by a curved and discontinuous but sharp scarp that borders a ∼200 m wide and ∼500 m long depression interrupting the regular sub‐horizontal top of the hill; (b) the presence of scarps and trenches crosscutting the sub‐horizontal continental strata and a characteristic double‐crested line topographic morphology typical of DGSDs (Figure 2a); and (c) the described landforms only affect the middle sector of the Alto di Cacchia, as no other lineaments or scarps affect the hill toward SE.

#### 2.1.2 Sant'Erasmo DGSD

Sant'Erasmo DGSD is located just northwest of the Roccapreturo village, in the Middle Aterno River Valley. The sliding of the rock‐mass along the NW‐SE oriented major scarp (<1 km long) produced an about 500 m wide depression on the top of the unstable slope associated with a series of uphill facing scarps (Figure 2b). The lower one (<400 m long) affects the western flank of the Mt. Acquaro, located in the footwall of the Roccapreturo normal fault segment (Figure 2b), the longest segment of the ∼30 km‐long Middle Aterno Valley‐Subequana Valley fault system (Falcucci et al., 2011, 2015; Figure 1). The fault is about 10 km long along‐strike, the estimated Quaternary displacement is about 270 m and the minimum slip rate and earthquake recurrence intervals range from 0.23 to 0.34 mm/year and 5,340 to 1,758 years, respectively (Falcucci et al., 2015). A small depression (<100 m wide) filled by Pleistocene‐Holocene sub‐horizontal breccias with pink‐orange matrix, overlying Early Cretaceous platform carbonates (“Calcari a Rudiste e Orbitoline” fm.) dipping at ∼25° to NE, is associated with this counterslope scarp (Figure 2b). The large depression associated with the major scarp and the counterslope scarp partially arresting the sliding of the rock‐mass have been interpreted as a morphological feature that indicates the presence of DGSD.

#### 2.1.3 Mt. Serrone DGSD

Mt. Serrone DGSD, located just northeast of Gioia dei Marsi village, is a gravitational rock‐mass deformation accommodated by several scarps affecting the western slope of Mt. Serrone (∼1,350 m a.s.l.). The major scarp accommodating the DGSD dips to NE, antithetically with respect to the San Benedetto‐Gioia dei Marsi normal fault segment. The latter, which is part of the Fucino seismogenic fault system, borders the south‐eastern sector of the Fucino Basin (Figures 1 and 2c). The depression associated with the DGSD (<400 m wide) is filled with colluvial and talus deposits resulting from the erosion of the Cretaceous carbonates (average dip angles of ∼20° to NE) of Mt. Serrone. The carbonate rocks are juxtaposed with the Val Roveto flysch by the thrust bordering the eastern side of Giovenco Valley (Figure 1; Vezzani & Ghisetti, 1998). Here, several morphological features indicative of a DGSD, such as scarps and counterslope scarps, slope‐parallel trenches, open fractures, and alignments of NW‐SE oriented small depressions have been identified through photogeological and field analyses (Moro et al., 2009, 2012).

#### 2.1.4 Colle Cerese DGSD

“Colle Cerese” hill (∼1,100 m a.s.l.) is located just northeast of Venere village. An impressive double‐crested ridge interrupts the regular morphological slope continuity of the hill toward the Fucino Basin (Figure 2d). San Benedetto‐Gioia dei Marsi fault segment (SGf, in this area called Venere sector) crops out at the base of the slope. The normal fault activity resulted in the uplift of the footwall and in its gravitational instability (Moro et al., 2009; Stramondo et al., 2005). The SGf (about 10 km long along‐strike) reactivated at the same time of the “Marsicana Highway” fault and other faults belonging to the Fucino fault system during the 1915 *M*_w_ = 7.1 Avezzano earthquake (Rovida et al., 2020). The estimated maximum throw attributed to the SGf ranges between 800 and 1,300 m (Cavinato et al., 2002; Roberts & Michetti, 2004), suggesting a minimum slip rate of 0.24–0.29 mm/year (Galadini & Galli, 1999). Colle Cerese hill is carved into Cretaceous platform carbonates, juxtaposed by the SGf with the Quaternary lacustrine deposits filling the Fucino Basin (Vezzani & Ghisetti, 1998). The large depression between the two ridge crests (>1 km long and ∼400 m wide) is filled with Pleistocene‐Holocene fluvio‐lacustrine and talus deposits (Moro et al., 2009; Figure 2d). The double‐crested ridge morphology is a clear evidence of a DGSD affecting Colle Cerese hill. Other evidences of gravitational deformation, such as counterslope scarps, slope‐parallel trenches, and open fractures, were described in Moro et al. (2012).

#### 2.1.5 Valle Force Fault (Normal Fault Bordering a Small and Narrow Basin)

Valle Force fault (<2 km in length along‐strike) is one of the several NE dipping normal faults bordering small and narrow intermontane basins located in the area between Campo Imperatore basin, to NE, and Middle Aterno Valley, to SW (D'Agostino et al., 1998; Falcucci et al., 2015; Galadini & Messina, 2004; Figure 1). This “Basin and Range” like area consists on NE dipping (dip angles of 25°–40°) antithetic normal faults bounding southward the tilted blocks that likely detach onto a splay of the Gran Sasso thrust at relatively shallow depth (∼2 km). The presence of a shallow‐seated detachment fault is supported by the very limited dimensions of the blocks tilted by the fault (D'Agostino et al., 1998; Falcucci et al., 2015). Valle Force fault is located just north‐east of Barisciano village (Figure 2e). The fault juxtaposes the Mesozoic platform carbonates (“Calcari a Coralli e Diceratidi” fm.) forming the footwall ridge (∼1,200 m a.s.l.) with Pleistocene‐Holocene colluvial deposits and Lower Pleistocene slope‐derived calcareous breccias with pink matrix (“Brecce Mortadella” in Demangeot, 1965 or “Brecce di Fonte Vedice” in Bosi & Messina, 1991). The deposition of the “Brecce Mortadella” was coeval to the normal activity of the fault (D'Agostino et al., 1998).

## 3 Methods

Photogeological and geomorphological analyses of the study area allowed us to identify peculiar structural features associated with DGSDs. The latter were investigated on the field by measuring the fracture network affecting the host rocks in the footwall of the major slip surfaces. We distinguished joints and shear fractures from open fractures or fissures and measured the attitude and kinematics (i.e., rake) of the major slip surfaces when possible. The structural data were reported in topographic maps at 1:1,000 scale, produced by exploiting the aerial photos (spatial resolution of 10 × 10 m) provided by the Abruzzi Geoportal (http://geoportale.regione.abruzzo.it/Cartanet) and plotted as poles into a stereonet (Schmidt equal area, lower hemisphere). In the case of Alto di Cacchia, Sant'Erasmo and Colle Cerese DGSDs, and of the Valle Force fault, we also used high‐resolution orthomosaics produced by stitching hundreds of pictures (we used Agisoft Metashape Pro and Pix4D software) taken at 100–150 m from the ground with a drone (Phantom 4 Advanced and MAVIC 2 Pro). With regards to rock samples, syton‐polished thin sections of the slip zones associated with the major and secondary slip surfaces have been produced by cutting the samples perpendicular to the slip surface and parallel to the slip direction (where recognizable, otherwise along the dip direction). The thin sections were photo‐scanned at high resolution (4,000 dots per inch) both in plane and in cross polarized nicols and then observed under the optical microscope (OM). The scans of the thin sections were edited using specific tools by Adobe Photoshop (Ps) to highlight the shape of the clasts, the presence of minor fractures and veins, and the texture of the fine matrix surrounding the clasts.

Most of the thin sections were investigated for microstructural analysis with the scanning electron microscope (SEM) CamScan MX3000 (resolution 300 nm in back‐scatter electrons) installed at “Dipartimento di Geoscienze” (Padua Univ.) and with the Field Emission SEM (FESEM) Merlin Zeiss (resolution 200 nm in back‐scatter electrons) installed at the CERTEMA laboratory (Grosseto, Italy). Pictures were taken in backscattered electron (BSE) mode with an acceleration voltage of 8–10 kV and at a working distance of 4.7–6.1 mm.

## 4 Results

### 4.1 Structural Architecture

**Figure 3:**
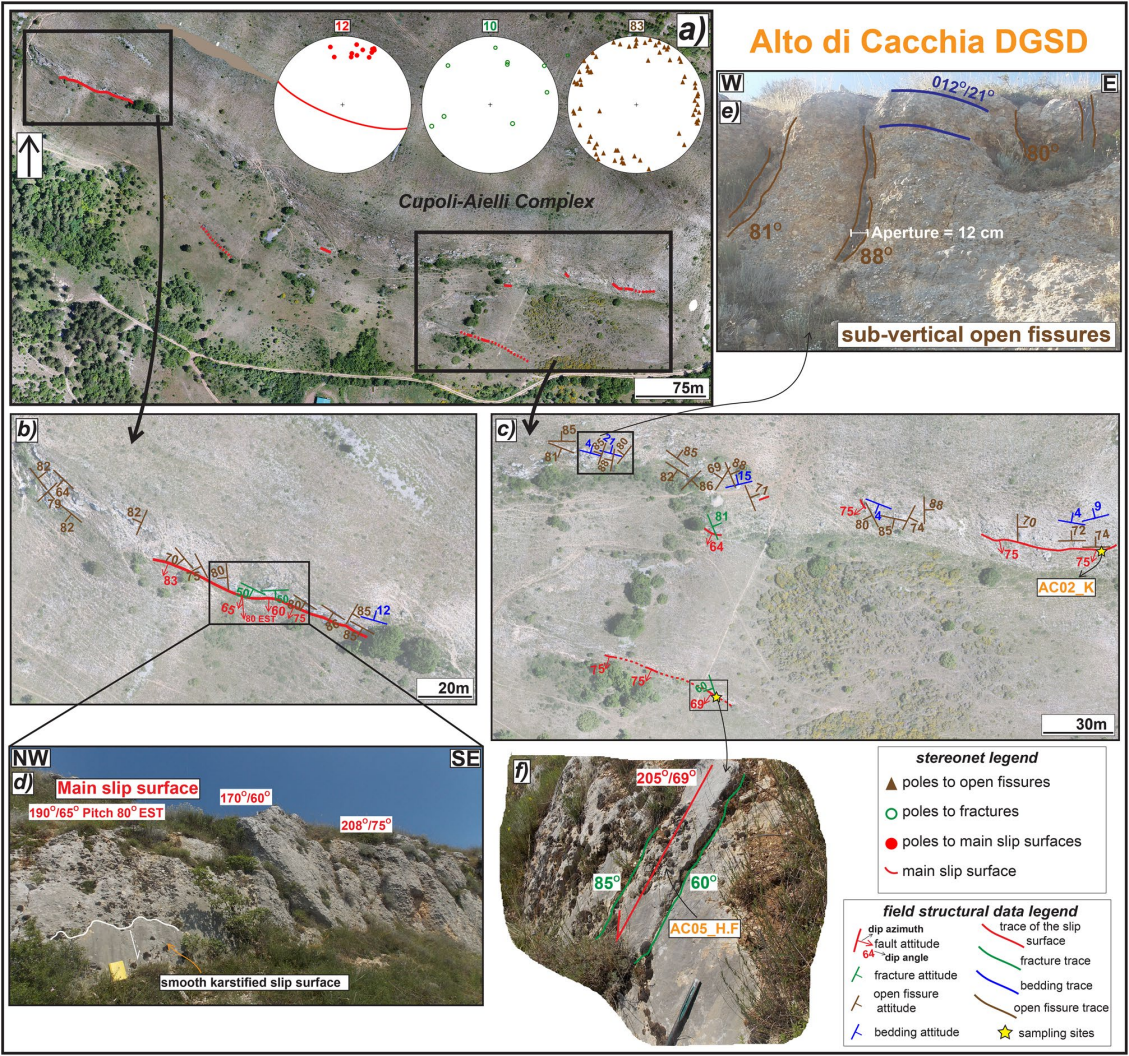
Structural sketch map of Alto di Cacchia deep‐seated gravitational slope deformation (DGSD). (a) Orthomosaic of the major and secondary scarps crosscutting the flat top hill carved into Pleistocene conglomerates. (b) Zoom on the north‐western tip, where the major karstified scarp outcrops for ∼100 m of length along‐strike, locally appearing smooth (d). (c) Zoom on the south‐eastern sector, where a secondary hangingwall sharp scarp outcrops for ∼50 m, including patches of Holocene sediments leant on the surface (f) (Sample AC05_H.F). (e) Sub‐vertical open fissures crosscutting sub‐horizontal conglomeratic strata; the surfaces delimiting the fractures partially mimic the morphology of the largest pebbles. The attitude of the structural data is reported as dip azimuth/dip angle or only as dip angle.

In this section, we present structural data (i.e., fault and fractures) collected in the footwall of the five case studies over the entire length of the major scarp. Fractures are distinguished in: (a) fractures *sensu strictu* or joints (i.e., extensional fractures that do not exhibit noticeable shear displacement between the fracture surfaces), shear fractures (i.e., small mesoscale fractures accommodating very limited displacement parallel to the fracture surfaces, commonly arranged as conjugate pairs, and with no visible damage zone in the field) and hybrid fractures, a combination of the first two types (Engelder, 1987; Fossen, 2010; Pollard & Aydin, 1988); (b) slip surfaces or sharp scarps with associated slip zone and damage zone beneath; and (c) open fractures or fissures (i.e., joints with >1 cm of aperture between the two opposite fracture surfaces), locally filled by unconsolidated soil deposits (e.g., Figures 3e, 5f and 6e). The spatial arrangement of joints, shear fractures, hybrid fractures, and fissures allowed us to assess the thickness of the footwall damage zone and to infer the eigenvectors of the stress tensor.

#### 4.1.1 Alto di Cacchia (DGSD Affecting Pleistocene Deposits)

Alto di Cacchia DGSD is limited by a ∼500 m long and discontinuous sharp scarp dipping on average ∼70° and crosscutting sub‐horizontal (dip angles from 4° to 15°) conglomerates mainly dipping toward NNW‐NNE (Figures 2a and 3a–3e). The scarp is karstified and locally smoothened by the rainwater flow. Close to the south‐western tip the scarp is about 1 m high, whereas in the north‐western tip, the scarp has a lateral continuity of ∼100 m and is up to 3 m high (Figures 3b and 3d). Here, the slip surface is sharp with some more polished patches, and shows slickenlines plunging 80° East (almost pure normal dip‐slip, Figure 3d). Two smaller scarps, less than 1 m high and ∼50 m long, outcrop discontinuously within the upper depression of the DGSD (Figures 3c and 3f). Patches of less cemented Holocene deposits filling the upper depression lean on these scarps and locally preserve them from weathering (Figure 3f).

The sub‐horizontal cemented conglomerates outcropping in the footwall of the major scarp are cut by fissures (from 1 to 12 cm of aperture) dipping >70° and, to a less extent, by fractures. The strike of the open fissures is very scattered (stereonets in Figure 3a) and the surfaces of the largest fractures locally mimic the shape of the largest pebbles of the conglomerate (Figure 3e). The smallest open fractures are filled by recent unconsolidated soil deposits.

#### 4.1.2 Sant'Erasmo (DGSD in the Footwall of a Main Seismogenic Fault)

**Figure 4:**
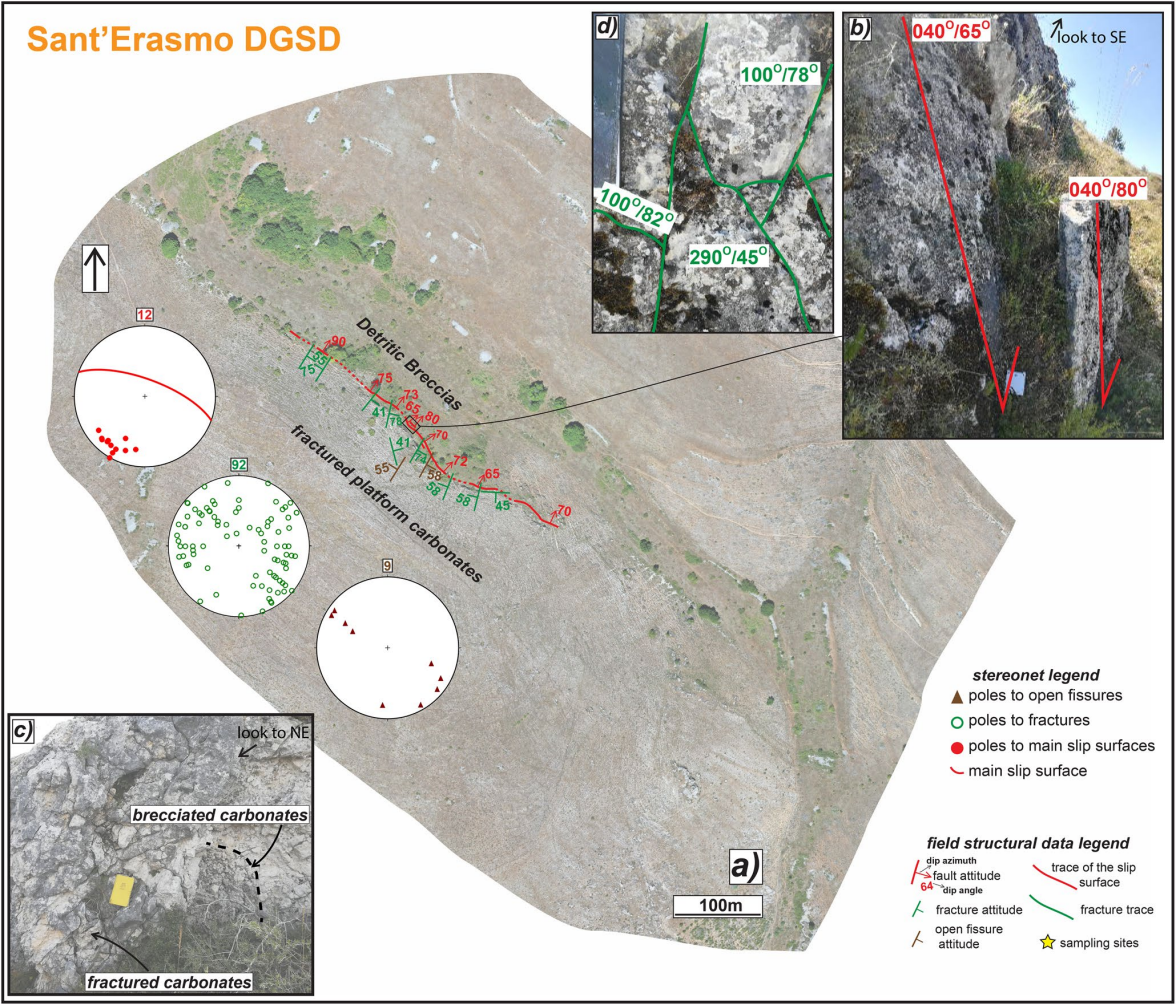
Structural sketch map of the counterslope scarp delimiting to SW the Sant'Erasmo deep‐seated gravitational slope deformation (DGSD). (a) Orthomosaic of the scarp juxtaposing Cretaceous fractured limestones with Pleistocene‐Holocene detritic breccias. (b) Major sharp scarp with associated a secondary sub‐vertical hangingwall scarp crosscutting the breccias and forming a gravitative trench. (c) Fractured footwall carbonates, locally appearing as brecciated. (d) Conjugate shear fractures affecting the major slip surface. The attitude of the structural data is reported as dip azimuth/dip angle.

Sant'Erasmo DGSD is located in the footwall of Roccapreturo seismogenic normal fault segment and is limited by a ∼700 m long major scarp striking NW‐SE, sub‐parallel, and synthetic to the Roccapreturo fault (Figure 2b). The field work was conducted on the small counterslope scarp (<400 m long along‐strike and up to 3 m high) affecting the eastern slope of Mt. Acquaro and bordering a small depression filled by Pleistocene breccias and colluvial deposits (Figures 2b and 4a). The counterslope scarp is strongly karstified and displaces Cretaceous platform carbonates with average dip angles of ∼70°. In the hangingwall of the scarp, small secondary sub‐vertical sharp scarps cut the Pleistocene breccias forming small trenches (Figure 4b). In the footwall, the platform carbonates are intensely fractured (in some parts brecciated; Figure 4c) by joints and shear fractures and, to a less extent, by open fractures (tens of centimeters long and up to 5 cm of aperture; stereonets in Figure 4a). The attitude of both fractures and open fissures is scattered with dip angles ranging from 88° to 15° (Figure 4a). However, most of the shear fractures are arranged in two main conjugate sets striking ca. NE (Figures 4a and 4d).

#### 4.1.3 Mt. Serrone (DGSD in the Footwall of a Main Seismogenic Fault)

**Figure 5:**
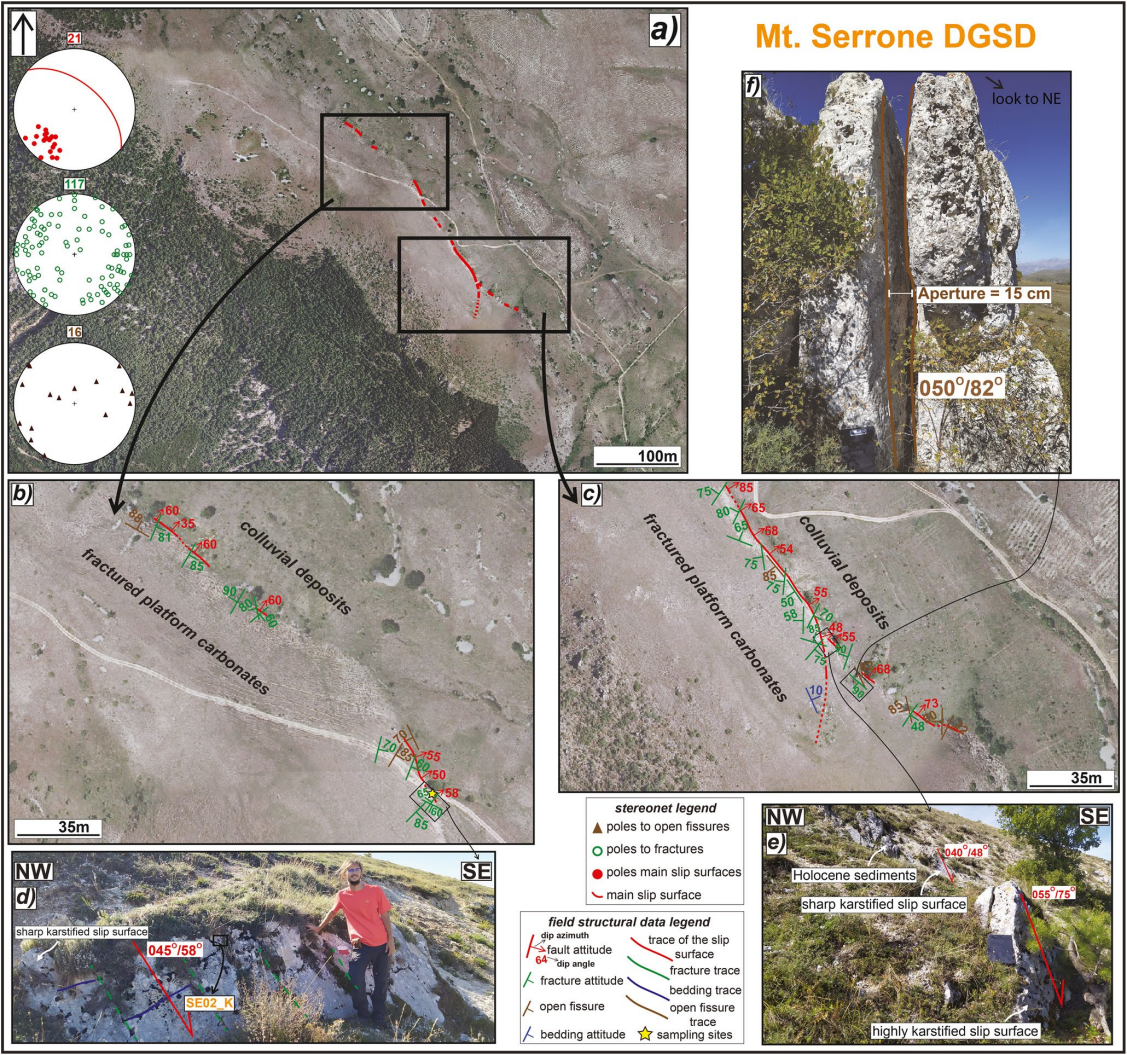
Structural sketch map of Mt. Serrone deep‐seated gravitational slope deformation (DGSD). (a) Orthomosaic of the major scarp affecting the north‐eastern slope of Mt. Serrone, carved into Mesozoic platform carbonates. (b) Zoom on the middle to north‐western portion. (c) Zoom on the middle to south‐eastern portion, where the scarp dextrally steps. (d) Detail of the sharp scarp in the middle sector cutting NE dipping carbonates with dip angles of ∼45° (Sample SE02_K). (e) Dextral step between the N‐S oriented sharp scarp, locally covered by patches of Holocene cemented sediments, and the much more karstified NW‐SE oriented scarp. (f) Large sub‐vertical fissure affecting the carbonate rocks in the step‐over zone. The attitude of the structural data is expressed as dip azimuth/dip angle.

This DGSD affects the eastern slope of Mt. Serrone and is limited by a ∼500 m long sharp scarp displacing sub‐horizontal Mesozoic platform carbonates antithetically with respect to the San Benedetto‐Gioia dei Marsi normal fault (Figures 2c and 5a–5c). In the middle sector, the scarp is exposed continuously for ∼200 m along‐strike and up to a maximum height of ∼3 m and dip angles of ∼50° (Figures 5a and 5d). Toward south‐east, the strike of the scarp rotates from NW‐SE to NNW‐SSE and steps dextrally to SE. The right‐stepped segment is more karstified and dips of ∼65° to the NE (Figure 5e), similarly to the north‐eastern termination of the scarp. Patches of Holocene poorly cemented deposits filling the upper depression of the DGSD are leant on the slip surface of the left stepped segment (Figure 5e). In the relay zone between the N‐S and NW‐SE oriented scarps, several meter‐long sub‐vertical fissures (up to 15 cm of aperture) crosscut the carbonate rocks (Figure 5f). As for the Sant'Erasmo DGSD case, the shear fractures are arranged into conjugate sets striking at N280°–330° with scattered dip angles (from sub‐vertical to sub‐horizontal: stereonets in Figure 5a).

#### 4.1.4 Colle Cerese (DGSD in the Footwall of a Main Seismogenic Fault)

**Figure 6:**
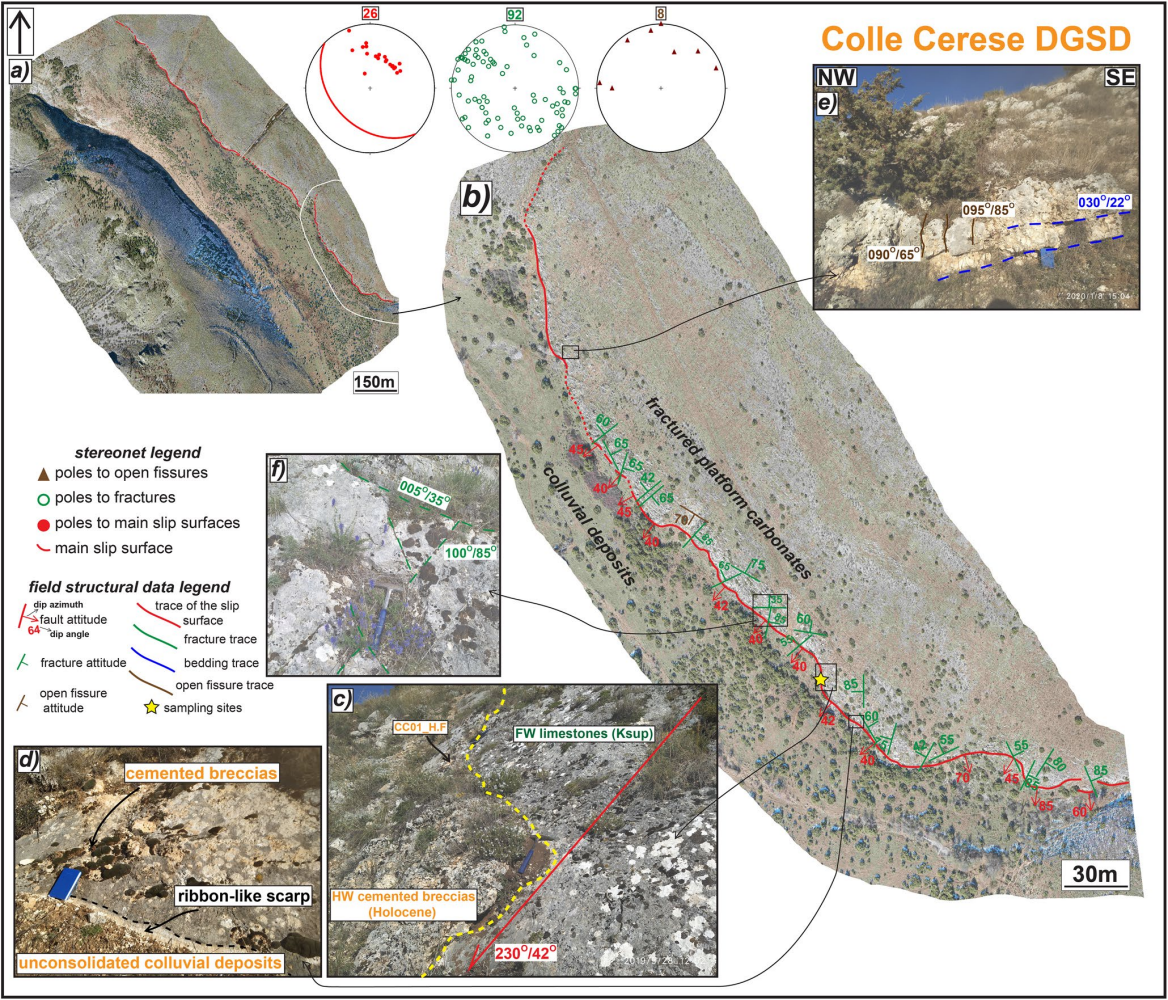
Structural sketch map of Colle Cerese deep‐seated gravitational slope deformation (DGSD). (a) Orthomosaic of the major scarp affecting the south‐western slope of Cerese Hill. (b) Zoom on south‐eastern sector, where the sharp scarp juxtaposes Cretaceous platform carbonates with Holocene breccias and colluvial deposits with average dip angles of ∼45° (c) (Sample CC01_H.F). (d) Detail of the sharp slip surface, locally preserved by patches of cemented breccias, with ∼5 cm thick fresh exposure (i.e., ribbon‐like scarp). (e) Sub‐vertical fissures crosscutting the sub‐horizontal carbonate‐built strata in footwall. (f) Conjugate shear fractures affecting the slip surface. The attitude of the structural data is reported as dip azimuth/dip angle.

Colle Cerese DGSD is located in the footwall of San Benedetto‐Gioia dei Marsi normal fault segment, in the Venere sector, and is limited by an about 1.5 km long sharp scarp striking NW‐SE (Figures 2d and 6a). The field work was conducted in the south‐eastern sector of the scarp (Figure 6b). Here, the major slip surface juxtaposes Mesozoic platform carbonates with Pleistocene colluvial deposits and unconsolidated breccias filling the large depression. The scarp dips to SW with dip angles of ∼45°, locally reaching over 10 m of height (Figure 6c). Patches of more cemented hangingwall breccias are leant on the slip surface and locally preserve it from weathering (Figures 6c and 6d). A 4–5 cm thick white in color fresh scarp exposure is locally recognized (i.e., “*nastrino*” or ribbon‐like scarp; Figure 6d). The sub‐horizontal carbonate strata are intensely fractured close to the major scarp, but if analyzed at a distance of tens of meters away, they are cut by few small sub‐vertical joints and open fractures (Figure 6e). As in Sant'Erasmo and Mt. Serrone DGSDs, fractures are arranged in conjugate sets striking N290°–340° with scattered dip angles (from sub‐vertical to sub‐horizontal: see stereonets and Figure 6f).

#### 4.1.5 Valle Force (Minor and Vertically Confined Normal Fault)

**Figure 7:**
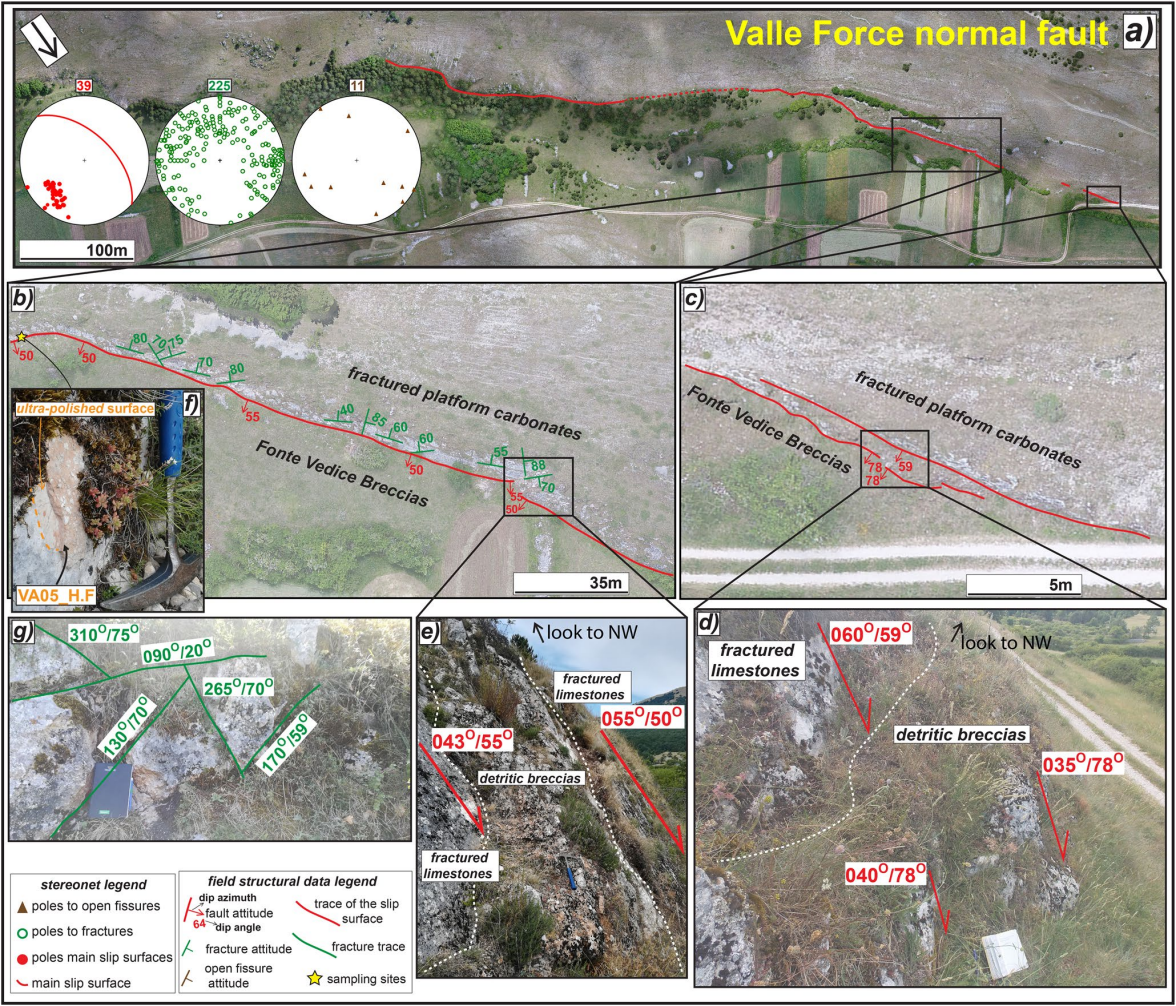
Structural sketch map of Valle Force normal fault. (a) Orthomosaic of the main scarp juxtaposing Cretaceous limestones with Lower Pleistocene breccias and Holocene deposits. (b) Zoom on the sharp fault scarp where it steps dextrally involving the hangingwall breccias in the relay zone (e). (c) Zoom on the north‐western tip, where secondary high angle scarps crosscut the hangingwall breccias (d). (f) Patch of hangingwall breccias on the slip surface. The latter appears as ultra‐polished where the breccias are removed (sample VA05_H.F). (g) Conjugate shear fractures affecting the fault scarp with different attitude. The attitude of the structural data is reported as dip azimuth/dip angle.

Valle Force normal fault has a very sharp scarp constantly outcropping for about 1.5 km along‐strike, up to 8 m of high in the middle sector, with dip of ∼50° (Figures 7a, 7c and 7e). The fault juxtaposes Cretaceous platform carbonates with colluvial deposits filling the basin (Pleistocene‐Holocene in age) and Lower Pleistocene calcareous breccias. Close to the north‐western tip, secondary hangingwall scarps dipping to SW with dip angles of 60°–80°, sub‐parallel to the major one, displace the Pleistocene breccias, possibly associated with a gravitational instability (Figure 7d). Just south‐east along‐strike, the fault scarp steps dextrally and deforms the breccias in the relay zone (Figure 7e). In the middle sector of the fault, patches of the hangingwall breccias cover the major scarp, thus preserving the fault core (Figure 7f). Where the breccias are removed, the fault surface is ultra‐polished (Figure 7f). As for Sant'Erasmo, Mt. Serrone, and Colle Cerese DGSDs, a large number of joints and shear fractures cut the fault surface and the footwall carbonates that sporadically outcrops along the fault strike (stereonets in Figure 7a). Most of the shear fractures are arranged in conjugate sets dipping on average (dip angle/dip azimuth) 60°/N260°–300° and 60°/N130°–180° (Figure 7g). The dip angle of both shear fractures and open fissures is quite scattered, ranging from 80° to 45° (Figures 7a and 7g).

### 4.2 Microstructures of the Slip Zones

**Figure 8:**
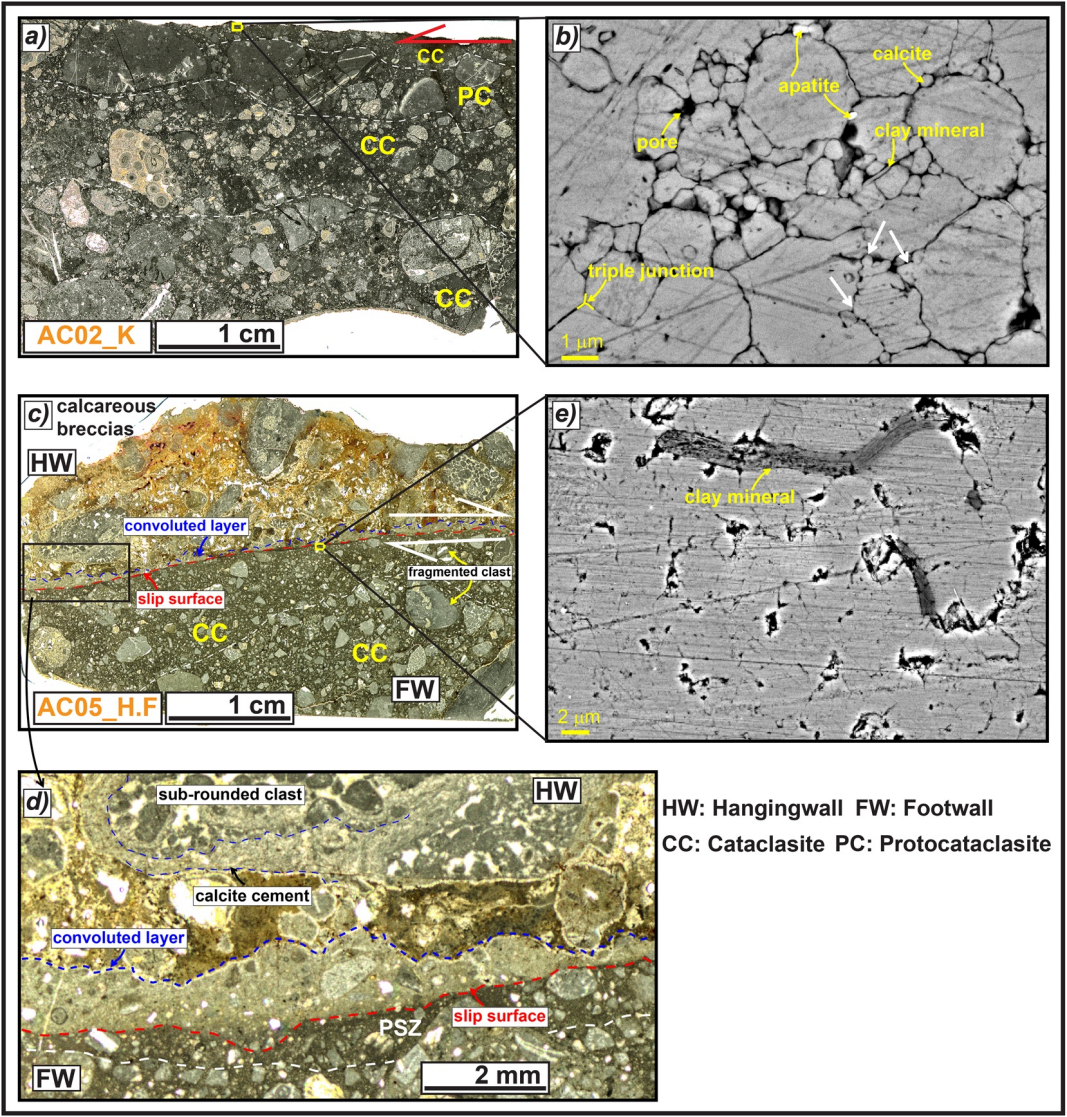
Microstructures of the slip zones relative to the major and secondary scarps of Alto di Cacchia deep‐seated gravitational slope deformation. (a) Slip zone of the major scarp, made of sub‐parallel cataclastic and proto‐cataclastic layers that are difficult to identify in the upper side because of the dark color at optical microscope of both fine matrix and larger clasts (sample AC02_K). (b) Scanning electron microscope (SEM) image of the fine matrix on the top, formed by packed calcite micro‐grains separated by sub‐micrometric in size pores and grains of apatite or clay minerals. The grain boundaries are straight, locally forming triple junctions, but at some points irregular, suggesting grain indentation (white arrows). (c) The wall of the secondary scarp includes a well‐developed cataclasite in the footwall and fracture‐free, cemented calcareous breccias in the hangingwall (sample AC05_H.F). (d) The footwall cataclasite includes a <1 mm thick principal slip zone (PSZ), separated by the hangingwall rocks by a 1–2 mm thick convoluted layer possibly produced by dissolution and precipitation‐cementation of the underlying PSZ. (e) SEM image of the matrix from the PSZ, composed of packed calcite micro‐grains with faint to straight grain boundaries and including clay minerals.

**Figure 9:**
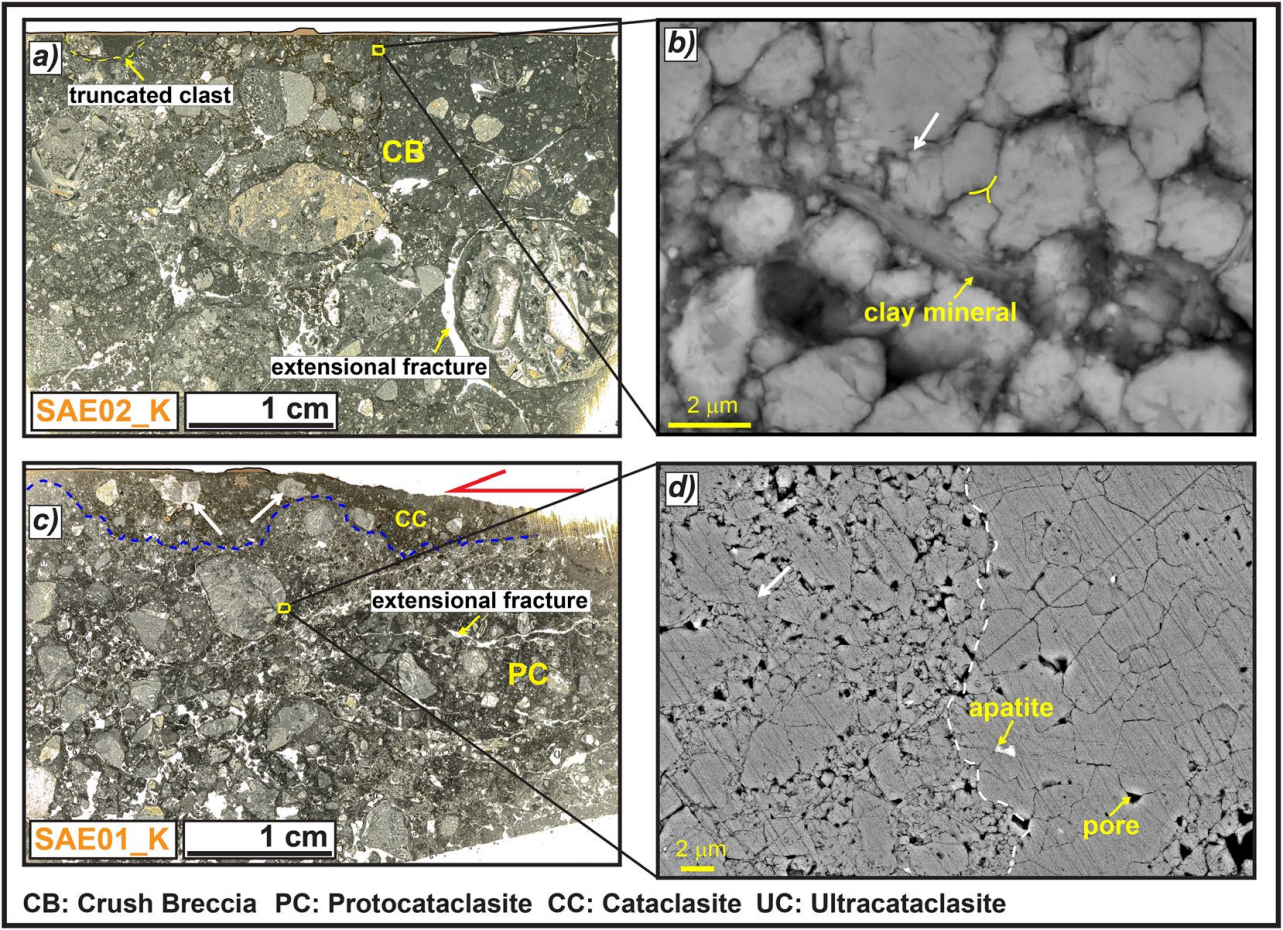
Microstructures of the slip zone of Sant'Erasmo deep‐seated gravitational slope deformation. (a) Thin section scan of the slip zone, a crush breccia formed by cm‐to‐mm in size rounded clasts (the slip surface was drawn because it was cut by the scanner) (sample SAE02_K). (b) Scanning electron microscope image of the hangingwall matrix filling the fractures right beneath the slip surface, composed of packed calcite micro‐grains, with straight to irregular boundaries (white arrow) and clay minerals partially filling the pore spaces. (c) Thin section scan of the slip zone where it is found, a cataclastic layer close to the slip surface due to the involvement of the hangingwall matrix during shearing, composed of <1 cm in size sub‐rounded clasts locally oriented with the long axis sub‐parallel to the slip surface (white arrows) (sample SAE01_K). (d) The porous matrix (left side to the dashed line) is composed of calcite micro‐grains with straight contacts, locally indented (white arrow), and is cut by veins of calcite, with pore spaces locally filled by apatite crystals (right side).

**Figure 10:**
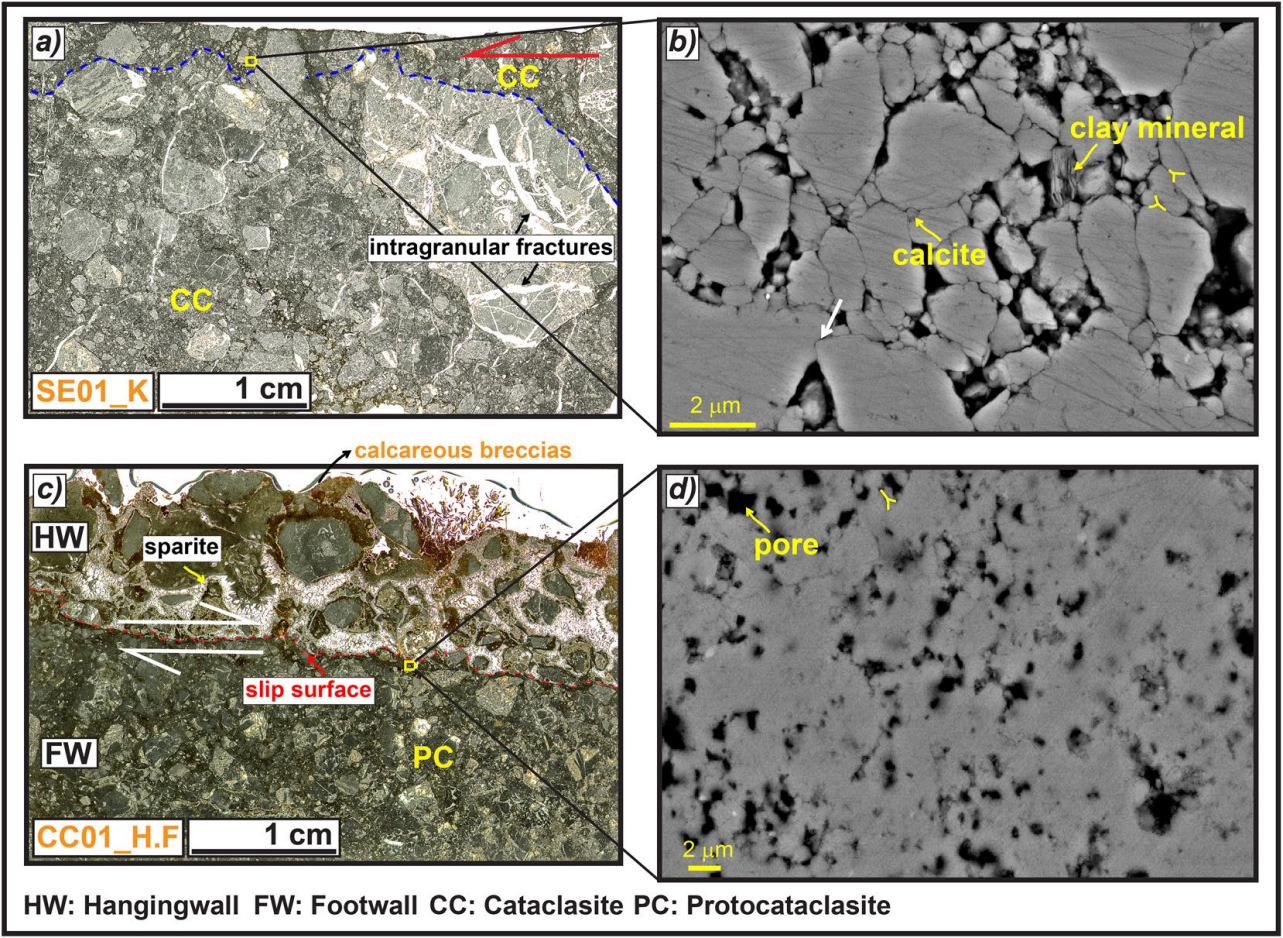
Microstructures of the slip zones of Mt. Serrone and Colle Cerese deep‐seated gravitational slope deformations (DGSDs). (a) The slip zone of Mt. Serrone DGSD has a cataclastic fabric composed of cm‐to‐mm in size angular clasts, internally fractured, surrounded by a dark‐gray fine matrix (sample SE01_K). (b) Scanning electron microscope image of the matrix close to the slip surface, composed of calcite micro‐grains, with straight to stylolitic‐like boundaries forming triple junctions and indentation structures (white arrow). The empty spaces among the grain boundaries are locally filled by clay minerals. (c) Thin section scan of the scarp wall of Colle Cerese DGSD: a quite rough slip surface delimits a proto‐cataclasite made of calcareous angular to sub‐rounded clasts in the footwall with Holocene calcareous breccias cemented by sparite in the hangingwall (sample CC01_H.F). (d) The fine calcite matrix right beneath the slip surface is very porous, probably due to weathering and biogenic activity occurred before the sealing of hangingwall deposits, but clast indentation and triple junctions between grains are still recognizable.

**Figure 11:**
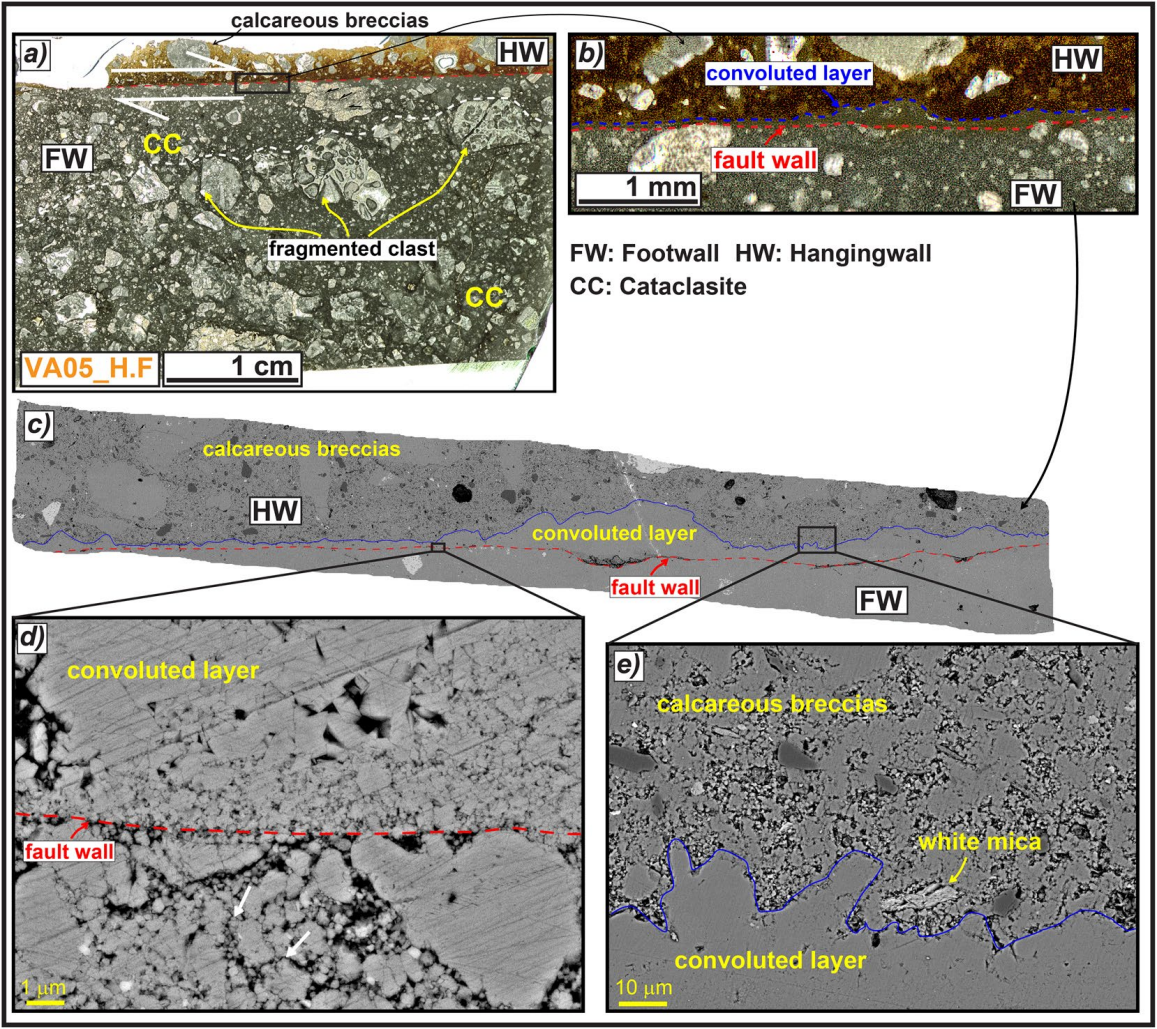
Microstructures of the Valle Force fault wall (sample VA05_H.F). (a) Thin section scan of the fault wall, composed of a well‐developed cataclasite in the footwall and calcareous breccias in the hangingwall. (b and c) Detail of the fault wall: the hangingwall rocks are separated from the footwall rocks by a <0.5 mm thick convoluted layer lying above the sharp slip surface, possibly formed by dissolution‐precipitation processes involving the footwall cataclasite. (d) Scanning electron microscope (SEM) image of the fault wall highlighting the clast indentation (white arrows) and triple junctions among grains at footwall. (e) SEM Image of the convolute contact between the dissolution‐precipitation front and the hangingwall breccias. The latter consists of angular clasts made of silica‐bearing minerals commonly oriented with the long axis sub‐parallel to the slip direction.

**Figure 12:**
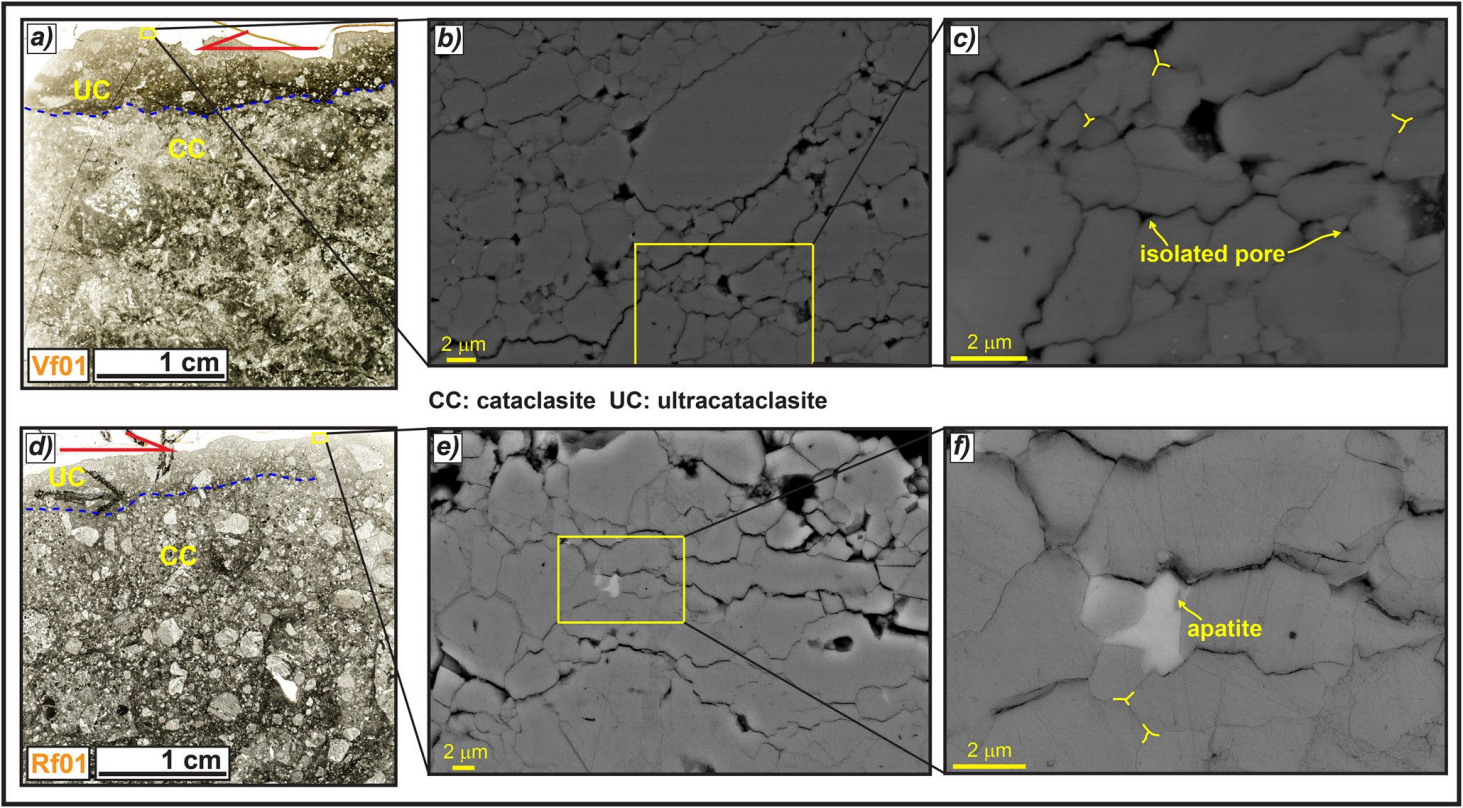
Microstructures of the slip zones relative to San Benedetto‐Gioia dei Marsi (in the Venere sector) and Roccapreturo seismogenic normal fault segments. (a) The slip zone of San Benedetto‐Gioia dei Marsi normal fault has a well‐developed cataclastic fabric and includes a well‐defined <0.5 cm thick cataclastic/ultra‐cataclastic layer on the top (sample Vf01). (b and c) Scanning electron microscope (SEM) images of the matrix close to the top, formed by highly packed calcite micro‐ to nanograins with straight to stylolitic‐like contacts, locally forming triple junctions and few isolated pores. (d) The slip zone of Roccapreturo fault has a similar fabric and also includes an ultra‐cataclastic level on the top (sample Rf01). (e and f) SEM images of the matrix from the ultra‐cataclastic level, formed by highly packed calcite micro‐grains with straight to irregular contacts separated by small pores, locally filled by apatite crystals.

The microstructures observed in the slip zones associated with the five selected field cases are described below following the fault rocks classification of Sibson (1977; Figures 8, 9, 10, 11). In addition, the microstructures of the slip zones of two major fault scarps of San Benedetto‐Gioia dei Marsi and Roccapreturo large normal faults are used as comparison (Figure 12). We define as slip surfaces the exposed karstified scarps and the scarps preserved by the Quaternary hangingwall sediments. The slip zones are the deformed rocks, up to several centimeters thick, located beneath the slip surfaces. Slip zones accommodate the shear strain produced during fault slip; PSZs are texturally distinct layers, usually <1 cm thick, located in the slip zone immediately beneath the slip surface. PSZs accommodate most of the fault displacement (Sibson, 2003).

#### 4.2.1 Alto di Cacchia (DGSD Affecting Pleistocene Deposits)

The slip zone in the footwall of the major scarp of Alto di Cacchia DGSD has a cataclastic fabric made of sub‐parallel and several mm thick cataclastic and proto‐cataclastic layers (sample AC02_K, Figure 8a). The slip zone is composed of few cm in size sub‐rounded clasts and several mm in size angular‐to‐rounded particles, locally fractured, immersed in a dark fine matrix. Close to the slip surface, a ∼2 mm thick and discontinuous cataclastic layer made of <1 mm in size angular to sub‐rounded grains was identified (Figure 8a). Under the SEM, the fine dark matrix of this layer is mostly composed of up to 5 μm in size calcite grains with straight boundaries forming triple junctions or separated by sub‐micrometric in size pores and grains of apatite or clay minerals with euhedral habit (Figure 8b). However, some calcite grain boundaries have irregular and stylolitic‐like aspect, suggesting grain indentation (white arrows in Figure 8b).

The slip zone located in the footwall of the secondary hangingwall scarp has a more “mature” cataclastic fabric (sample AC05_H.F). In fact, sub‐rounded and fractured mm in size clasts and sub‐mm in size angular clasts of the footwall rocks are dispersed in a dark‐brownish in color fine matrix that becomes more abundant (>60% in volume) toward the slip surface (Figure 8c). The latter is slightly undulated and has a sharp contact with the underlying calcite clasts (Figures 8c and 8d). The PSZ right beneath the slip surface is about 1 mm thick and made of sub‐millimetric in size clasts immersed in a darker fine matrix (Figure 8d). The matrix of the PSZ is composed of packed calcite micro‐grains with faint to straight grain boundaries forming triple junctions and by few clay minerals (SEM image, Figure 8e). The hangingwall rocks are separated from the footwall rocks by a 1–2 mm thick and continuous gray in color layer made of sub‐millimetric in size calcite grains partially dissolved by karst processes and possibly deriving from the underlying PSZ (Figure 8c). This origin of the calcite grains is also suggested by the convoluted contact of the PSZ with the hangingwall rocks. The latter are formed by dm‐to‐cm in size rounded carbonate clasts, almost fracture‐free, cemented by the precipitation of a porous and ochre in color matrix (Figures 8c and 8d). This microstructure suggests that the hangingwall rocks were cemented in situ and partially protected the scarp surface from weathering.

#### 4.2.2 Sant'Erasmo (DGSD in the Footwall of a Main Seismogenic Fault)

The slip zone located in the footwall of the counterslope scarp delimiting to SW, the Sant'Erasmo DGSD is a crush breccia to proto‐cataclasite formed by cm‐to‐mm in size rounded clasts immersed in a fine and porous matrix. The slip zone is cut by minor fractures sub‐parallel and sub‐orthogonal to the slip surface (samples SAE02_K and SAE01_K, Figures 9a and 9c). The fine reddish matrix of the hangingwall breccias fills the fractures located right beneath the slip surface and is part of a discontinuous cataclastic layer (<5 mm thick) located between the proto‐cataclasite and the slip surface (Figure 9c). This discontinuous layer is made of sub‐cm and sub‐rounded clasts with the long axis oriented sub‐parallel to the slip direction, immersed in a brownish ultra‐fine matrix (Figure 9c). The matrix is very porous and is mostly composed of sub‐micrometric to micrometric in size grains of calcite and clay minerals (Figure 9b). Calcite grains have straight boundaries, locally forming triple junctions, but indentations and sutured contacts are also observed (Figure 9b; left side of Figure 9d). The fine matrix is cut by calcite veins, with pore spaces locally filled by apatite crystals (right side of Figure 9d).

#### 4.2.3 Mt. Serrone (DGSD in the Footwall of a Main Seismogenic Fault)

Similar to the previous cases, the slip zone of Mt. Serrone DGSD has a cataclastic fabric composed of cm‐to‐mm in size angular to sub‐rounded clasts immersed in a dark ultra‐fine matrix (sample SE01_K, Figure 10a). The largest clasts are fractured and have a sharp contact with the slip surface that appears rough and karstified, suggesting possible dissolution by weathering processes (Figure 10a). The fine matrix is made of sub‐ to micrometer (>2 μm) in size calcite grains with straight to stylolitic‐like boundaries, locally forming triple junctions; indentation structures are very common (Figure 10b).

#### 4.2.4 Colle Cerese (DGSD in the Footwall of a Main Seismogenic Fault)

The main slip zone accommodating Colle Cerese DGSD is a proto‐cataclasite consisting of cm‐to‐mm in size fragmented angular clasts immersed in a dark in color ultra‐fine and porous matrix (<40% of the total volume; sample CC01_H.F, Figure 10c). Though in the matrix some sort of grain packing is still recognizable (clast indentation, grain boundaries forming triple junctions, etc.), the slip zone right beneath the slip surface is weathered, as suggested by the occurrence of pores possibly resulting from meteoric exposure and biogenic activities (Figure 10d). The slip surface is rough and covered by a Holocene calcareous breccia. The latter is formed by large in size (>5 cm) rounded pebbles cemented by a brownish to white in color calcite‐rich matrix and by sparite (Figure 10c).

#### 4.2.5 Valle Force (Relatively Small Normal Fault)

The slip zone in the footwall of Valle Force normal fault consists of a cataclasite similar to the one found in Alto di Cacchia DGSD (Figure 8c), but the slip surface is smoother and makes a sharp contact with the underlying clasts (sample VA05_H.F, Figures 11a and 11b). The cataclasite consists of <1 mm in size sub‐rounded clasts and few larger angular clasts (the latter with the long axis sub‐parallel to the slip surface) immersed in a fine matrix (Figure 11a). The amount of matrix increases approaching the slip surface. A <0.5 mm thick convoluted layer composed of both comminuted and packed calcite micro‐ to nanograins separates the footwall carbonates from the hangingwall breccias (Figures 11b and 11c). The convoluted contact with the hangingwall rocks reminds of a dissolution‐cementation front similar to the one observed in the scarp wall of Alto di Cacchia DGSD (Figures 8c and 8d). In general, the slip zone in footwall is formed by <1 μm to >5 μm in grain size calcite grains with evidence of clast indentation and rare triple junctions among grains (Figure 11d). The hangingwall breccias are composed of sub‐cm in size sub‐rounded to angular carbonate clasts cemented by a brownish porous and fine calcite‐rich matrix (Figures 11a, 11c and 11e). The matrix also includes silica‐bearing minerals such as quartz and micas with the long axis oriented sub‐parallel to the slip direction (Figure 11e). This preferential alignment and the size reduction of the grains toward the slip surface is indicative of the involvement of the hangingwall rocks in fault slip, but no evidence of mixing structures (e.g., injection or “fluidization” structures; Demurtas et al., 2016) between the hangingwall and footwall rocks was observed (Figures 11b–11d).

#### 4.2.6 San Benedetto‐Gioia dei Marsi and Roccapreturo Large Seismogenic Faults

In order to compare the slip zones found beneath the main slip surfaces of the DGSDs (Figures 8, 9, 10) and the Valle Force fault (Figure 11), we describe the main slip zones of two large slip seismogenic faults from the same area. San Benedetto‐Gioia dei Marsi (sample Vf01) and Roccapreturo faults (sample Rf01) are both about 10 km long segments of the Fucino and the Middle Aterno Valley‐Subequana Valley fault systems, and are capable of producing up to *M*_w_ 7.1 and *M*_w_ 6.5 earthquakes, respectively (Barchi et al., 2000; Falcucci et al., 2015) (see also Figure 14). The core measured in the Venere sector of San Benedetto‐Gioia dei Marsi fault is up to 1 m thick and includes several matrix‐ and cemented‐supported minor faults (Agosta & Aydin, 2006; Ferraro et al., 2018, 2019). The slip zone of the main fault is made of several cms thick cataclasite consisting of cm‐to‐mm in size sub‐rounded clasts immersed in a sub‐millimetric and dark in color ultra‐fine matrix (>50% in volume). The studied slip zones of the two faults lack of their respective slip surfaces. Nevertheless, both slip zones include a well‐defined, <0.5 cm thick, cataclastic/ultra‐cataclastic (matrix ca. 80%–90% in volume) layer approaching the (inferred) location of the slip surface (Figures 12a and 12d). The matrix includes few micrometers to tens of nanometers in size calcite grains with straight to stylolitic‐like boundaries and local grain indentation (Figures 12b and 12c). The grain boundaries locally form triple junctions and few isolated pores (Figures 12b, 12c and 12e), rarely filled by apatite crystals in the case of the Roccapreturo fault segment (Figure 12f).

## 5 Discussion

In this work, we have described (a) the fault/fracture network in the footwall of four DGSDs and also of a minor normal fault bordering a small depression (used as comparison) in the central Apennines (Section 4.1); and (b) the micro‐ to nanostructures of the slip zones of the major and secondary scarps associated with the selected case studies. The slip zones have been compared with those associated with two large seismogenic faults hosting DGSDs in their footwall (Section 4.2). In the following sections, we discuss (a) the formation and the reactivation of the fractures and slip surfaces associated with the DGSDs (Section 5.1), (b) the formation of DGSDs in the central Apennines (Section 5.2), and (c) the deformation mechanisms active in DGSDs hosted in carbonate rocks (Section 5.3).

### 5.1 Formation and Reactivation of Fault/Fracture Networks in DGSDs

**Figure 13:**
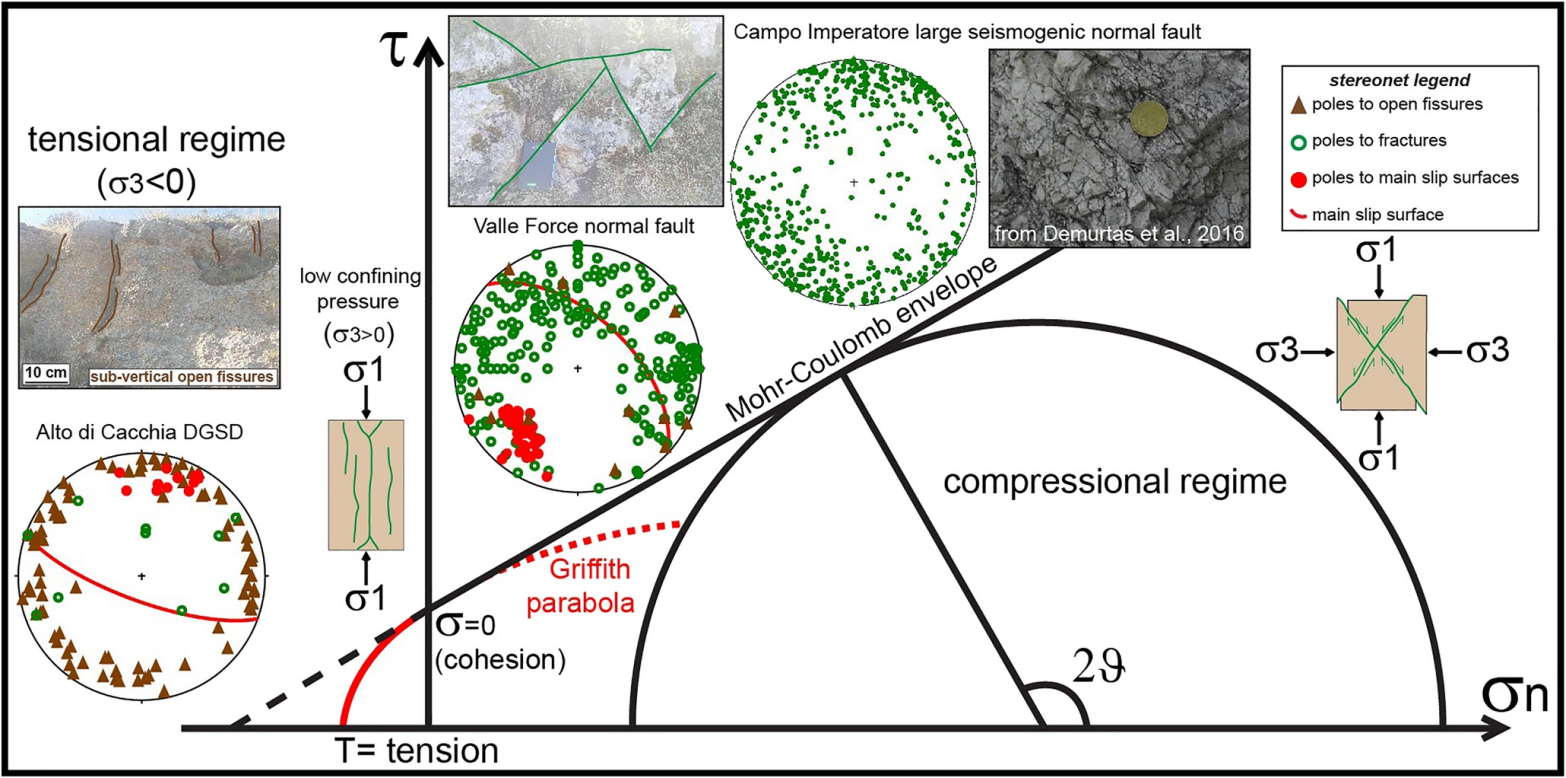
Stereonets showing the poles of the fractures affecting the footwall conglomerates of Alto di Cacchia deep‐seated gravitational slope deformation (DGSD), Valle Force small normal fault, and Vado di Corno seismogenic fault (Campo Imperatore fault system) and their relation with the orientation of newly formed fractures according to the Mohr‐Coulomb failure criteria in the Mohr space. The large number of fissures and joints dipping at >70° (“high angle”) cutting the footwall rocks of Alto di Cacchia DGSD were reasonably formed under a tensional regime or at very low confining pressures. The conjugate shear fractures affecting the footwall rocks close to the Valle Force fault scarp developed at higher confining pressures and differential stresses. Most of the fractures affecting Vado di Corno fault core strike NW‐SE, consistent with the accommodation of larger strains during the last extensional phase and deeper normal dip‐slip activity of the structure, exhumed from >1 km of depth.

**Figure 14:**
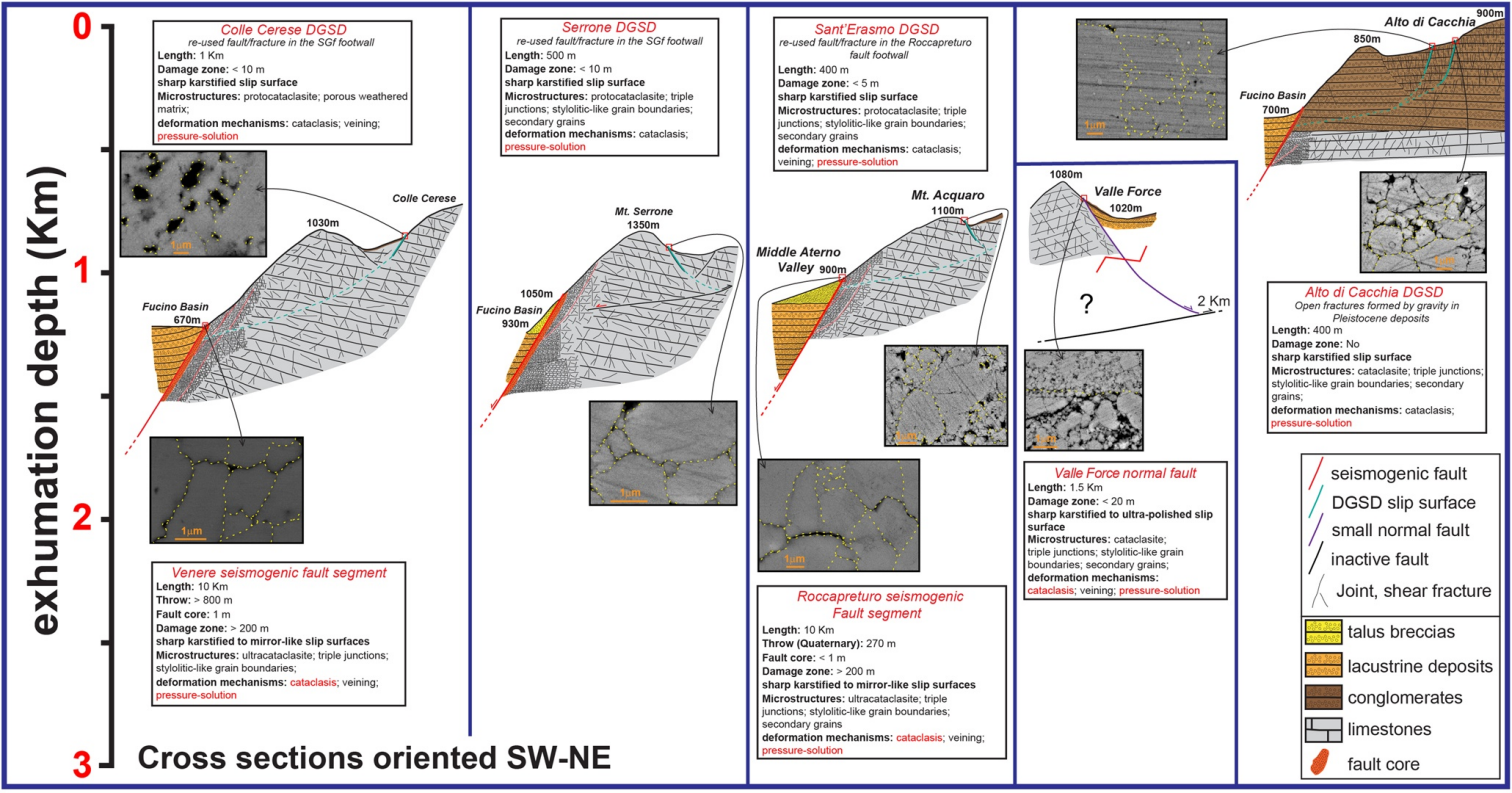
Geological cross sections showing the main characteristics of the studied cases (displacement of the slip surface, common microstructures associated, inferred deformation mechanisms, exhumation depth of the fault/fracture network, etc.). San Benedetto‐Gioia dei Marsi and Roccapreturo fault zones exhumed from >1 km of depth. Valle Force normal fault exhumed from shallower depths and flattens at about 2 km of depth on a preexisting thrust. The major slip surfaces of the deep‐seated gravitational slope deformations (DGSDs) reuse preexisting faults and fractures developed in the footwall of the associated large normal faults. The microstructures of the slip zones are very similar in both normal faults and DGSDs, suggesting cataclasis and low temperature diffusive processes (i.e., pressure‐solution) as the dominant deformation mechanisms active at shallow depth in carbonates.

The fault/fracture networks associated with the DGSD scarps suggest different loading conditions at the time of their formation. Based on the Mohr‐Coulomb failure criterion, the numerous sub‐vertical open fissures and joints crosscutting the sub‐horizontal conglomerates of Alto di Cacchia DGSD should have been developed in tensional regime or at very low confining pressures, with the maximum principal stress oriented sub‐parallel to the slip surface (Figures 13 and 14). As a matter of fact, open fractures and other tensional structures like gravitational trenches, ridge‐top grabens, and steep scarps commonly affect the upper and middle portions of DGSDs (Crosta et al., 2013; Esposito et al., 2007; Gori et al., 2014; Hungr et al., 2014). This interpretation is further supported by the observation that Alto di Cacchia DGSD affects the about 400 m thick Pleistocene Cupoli/Aielli Complex, which lies above Cretaceous carbonates. This would imply that the fault/fracture network formed at very shallow depth (*T* < 15°C and *P*_litho_ < 15 MPa, Figure 14). The scatter of the strike (but not of the dip angle) of the minor faults and fractures might be due to local stress rotations related to (a) the evolution of the syn‐sedimentary basin, and (b) exhumation process and hillslope evolution (e.g., with erosion and exhumation, one of the principal stresses may rotate to become normal to the slope surface).

In contrast, in the case of Sant'Erasmo (located in the footwall of the Roccapreturo main fault segment), Mt. Serrone, and Colle Cerese (located in the footwall of the San Benedetto‐Gioia dei Marsi main fault segment) DGSDs, the sub‐horizontal Cretaceous limestones in the footwall are cut by regularly spaced sets of joints and conjugate shear fractures that have a large scatter of both strike and dip angle (Figures 4, 5, 6). This spatial arrangement is consistent with the formation of these fractures at larger confining pressures with respect to the Alto di Cacchia and possibly under different stress fields from at least the Pliocene to the Quaternary (Figure 13).

Indeed, the fault/fracture network associated with these DGSDs is similar to the one of Valle Force small normal fault (Figures 7a and 13), that should flatten at 2–3 km of depth on a preexisting low angle fault (D'Agostino et al., 1998; Falcucci et al., 2015; Figure 14), and of the damage zones of large and seismogenic normal faults exhumed from 1 to 3 km depth (e.g., San Benedetto‐Gioia dei Marsi and Vado di Corno fault zones: Agosta & Aydin, 2006; Agosta & Kirschner, 2003; Demurtas et al., 2016; Fondriest et al., 2020; Figures 13 and 14). However, in the latter cases, most of the minor faults and fractures strike NW‐SE, consistently with the NE‐SW oriented Middle Pleistocene to Holocene stretching of the central Apennines (D'Agostino et al., 2011). In contrast, the few large sub‐vertical open fissures cutting the carbonate strata in the footwall of Colle Cerese, Mt. Serrone, and Sant'Erasmo DGSDs, and the absence of veins filling the fractures are consistent with a recent gravitational activity at shallow surficial conditions.

Therefore, most DGSDs in the central Apennines were interpreted by us as the result of gravity‐induced reactivation of preexisting minor faults or shear fractures located in the footwall of larger normal seismogenic faults, well‐oriented with respect to the actual stress field. Instead, Alto di Cacchia DGSD is the only studied DGSD that exploited fractures formed at a very shallow depth, given the above‐mentioned structural and stratigraphic constraints. Alto di Cacchia DGSD is accommodated by both newly formed fractures associated with the sliding of Pleistocene conglomerates, and possibly by the reactivation of minor faults and fractures produced by faulting at very shallow depths (200–300 m at maximum) (Figure 14).

The major scarps delimiting Alto di Cacchia, Mt. Serrone, and Sant'Erasmo DGSDs (<500 m long) are ∼3 m high (Figures 3, 4, 5), whereas those of Colle Cerese DGSD and Valle Force fault (>1 km in length) are locally up to 10 m high, respectively (Figures 6 and 7). Such large height values of the scarps are similar to those of most normal fault scarps outcropping in the central Apennines (up to 10 km in length along‐strike and accommodating up to 600 m of maximum throw; Ferraro et al., 2018). Moreover, the DGSD scarps appear very sharp although karstified, as well as most of the normal fault scarps cutting the carbonate rocks in the central Apennines (Agosta & Aydin, 2006; Galadini & Galli, 2000; Smeraglia, Bettucci, et al., 2017; Smeraglia, Billi, et al., 2017). This would imply that high and sharp scarps can be produced either by gravitational processes or by tectonic, possibly seismic, faulting, or by a combination of the two (Kastelic et al., 2017).

### 5.2 Formation of DGSDs in the Central Apennines

In the previous section we interpreted the major sharp scarps accommodating the studied DGSDs as preexisting (i.e., tectonic in origin) fault/fracture surfaces developed in the footwall of large seismogenic faults reactivated by gravitational hillslope deformation. According to this interpretation, the studied DGSDs develop, initially, from both preexisting and newly formed sub‐vertical fractures that spread from the hillslope surface toward the rock mass beneath. At this stage, fractures evolve into large fissures allowing the formation of ridge‐top grabens and gravitative trenches at the slope crest (Chigira, 1992; Gori et al., 2014; Mariotto & Tibaldi, 2015). Then, the spreading rock mass evolves into a large slide, due to the linkage of the sub‐vertical fissures and fractures propagating from the surface with preexisting minor faults and fractures in the footwall damage zone. The basal surface of the DGSD (i.e., Sackung‐type) flattens at few hundreds of meters depth possibly due to reactivation of preexisting low angle fractures/faults (e.g., P‐shear fractures or preexisting thrust faults) close to the master fault. The rotational sliding of the rock‐mass accommodated by the basal surface produces rock topples, trenches, and uphill‐facing scarps in the middle sector of the slope (Agliardi et al., 2001; Chigira, 1992). In the lower sector of the DGSD, the increase of the compressional forces at the toe causes the bulging of the rock‐mass (Chigira, 1992; Hermann et al., 2000; Mariani & Zerboni, 2020).

Unfortunately, and as for most of the Sackung‐type DGSDs worldwide, the main slip surface is less defined in the basal sector and is also buried by the displaced rock‐mass that cumulates at the base of the DGSD (Cruden & Varnes, 1996). Colle Cerese and Sant'Erasmo are Sackung‐type DGSDs affecting carbonate strata dipping in the opposite direction of the slope (i.e., anaclinal slope), allowing the development of scarps, uphill‐facing scarps, and a large double‐crested ridge morphology (Moro et al., 2009; Figures 2 and 14). On the other hand, in Mt. Serrone DGSD case, the bedding dips in the same direction of the slope (i.e., cataclinal slope) and the double ridge is less developed. Here, a series of steep downhill‐ and uphill‐facing scarps delimiting small grabens and gravitative trenches affect the entire hillslope (Hermann et al., 2000; Mariani & Zerboni, 2020; Figures 2 and 14). This mechanism of formation of DGSDs is consistent with those proposed for Sackung‐type DGSDs worldwide, even though other important factors (e.g., lithology, tectonic setting, glacial retreat, etc.) influence the type, shape, and behavior of each DGSD (Panek & Klimeš, 2016).

### 5.3 Deformation Mechanisms in Carbonate‐Hosted DGSDs

The slip zones in the footwall of the DGSD and of Valle Force fault scarps show very similar microstructures, especially when observed at micro‐metric scale (Figures 8, 9, 10, 11). At this scale, the textures of the fine matrix are also similar to those found in the slip zones of San Benedetto‐Gioia dei Marsi and Roccapreturo seismogenic normal faults (Figure 12), suggesting the activation of similar deformation mechanisms. Here, we will relate the microstructures of the slip zones of both DGSDs and normal faults to the deformation mechanisms active on carbonate rocks at upper crustal conditions.

#### 5.3.1 Microstructural Organization of the Slip Zones

In active seismogenic normal faults, the bulk of displacement during co‐seismic slip is mainly accommodated by <1 cm thick PSZs (Chester & Chester, 1998; Chester et al., 1993; Power & Tullis, 1989; Sibson, 2003). The major slip surfaces of large San Benedetto‐Gioia dei Marsi and Roccapreturo seismogenic faults are associated with a 10–30 cm‐thick cataclastic slip zone (Ferraro et al., 2018) that includes a ∼0.5 cm thick ultra‐cataclastic layer (PSZ, Figures 12a and 12d). In addition, the slip zones of Vado di Corno fault surface (Campo Imperatore fault system) contain mixed clasts and gouges deriving from both the hangingwall Quaternary deposits and the footwall Mesozoic carbonates arranged in “fluidization structures” or injections from the PSZ into the wall rocks (see Figures 3c and 11c of Demurtas et al., 2016).

In contrast, the slip zones in the footwall of the scarps of Sant'Erasmo, Mt. Serrone, and Colle Cerese DGSDs have a proto‐cataclastic fabric and lack a well‐defined cataclastic/ultra‐cataclastic layer (i.e., the PSZ) toward the slip surface (Figures 9a, 10a and 10c). Moreover, the slip zones of Valle Force fault (Figure 11a) and of Alto di Cacchia DGSD (Figure 8c) are similar; indeed, they consist of a well‐developed footwall cataclasite delimited from the hangingwall breccias by a sharp slip surface. Nevertheless, the slip surface of Alto di Cacchia DGSD appears rougher than the one of Valle force fault. Furthermore, in both the slip zones of Colle Cerese and of Alto di Cacchia DGSDs “fluidization” or injections structures (which are indicative of mixing between the hangingwall and footwall rocks) were not observed and the hangingwall breccias appear as poorly deformed and cemented (Figures 8c and 10c). This would suggest a possible emplacement, and subsequent in situ cementation, of the Quaternary hangingwall rocks right after the exhumation of the scarp. On the contrary, the hangingwall breccias of Valle Force fault were involved in the sliding, as suggested by the grain size reduction toward the slip surface and by the orientation sub‐parallel to the slip surface of the long axis of the silicate‐built grains (Figures 11a, 11c and 11e). However, “fluidization” structures were not found, suggesting lower strains (or strain rates) with respect to the major seismogenic faults (Figures 11b and 11c).

In conclusion, at the slip zone scale, the major active seismogenic faults differ from DSGDs because of their well‐developed cataclastic fabric that includes extreme localization in ultra‐cataclastic layers, thick cataclasites sealed by a dense calcite‐vein network, and mixing of footwall and hangingwall materials. It seems that these features can be associated with the larger slip accommodated by these faults with respect to the DGSDs or to the small faults. Nevertheless, other than this interpretation, there is no clear evidence regarding the microstructural organization of the slip zones that allowed us to distinguish DGSDs from tectonic faults. Moreover, since we claim that DGSDs scarps exploit preexisting minor tectonic faults/fractures (Section 5.1), the microstructures observed in the DGSD slip zones could have been mainly produced by tectonic sliding, rather than gravitational sliding. Further studies need to be carried out to obtain better constraints on the exhumation depth of these fault rocks, as for example, clumped isotopes analyses of the matrix and cements within and close to the slip zones.

#### 5.3.2 Matrix of the Slip Zones

Cataclastic and ultra‐cataclastic layers and their associated slip surfaces are the result of extreme deformation in brittle faults. As a consequence, the associated microstructures, and in particular those relative to the fine matrix, may yield information on the deformation mechanisms active during faulting and DGSD.

The matrix close to the slip surface of Valle Force fault and of the four DGSDs is composed of calcite micro‐ to nanograins, with straight to stylolitic‐like grain boundaries forming locally triple junctions and isolated pores with widespread clast indentation (Figures 8, 9, 10, 11). The matrix of the ultra‐cataclastic layers of San Benedetto‐Gioia dei Marsi and Roccapreturo seismogenic faults is similar, though the grain boundaries among calcite grains are straighter, the triple junctions more widespread and the pore spaces smaller (compare Figures 8b, 9b, 10b, 10d, and 11d with Figures 12b and 12e). Although similar, this latter fabric differs from the typical foam‐like fabric produced in the laboratory by shearing at seismic velocities (i.e., >0.1 m/s) carbonate‐built fault gouges (Demurtas et al., 2019; Fondriest et al., 2013; Pozzi et al., 2019; Smith et al., 2013; Verberne et al., 2013). In these experimental studies, the main deformation mechanism proposed is grain boundary sliding aided by diffusion creep that should be activated at *T* > 550°C (De Paola et al., 2015; Demurtas et al., 2019; Pozzi et al., 2019). Instead, natural and experimental observations have shown that chemical compaction by pressure‐solution driven by fluid‐rock interactions is the main process of porosity loss in carbonates, in particular in calcite‐rich rocks (Croizè et al., 2013; Ferraro et al., 2019; Gratier et al., 2013, 2015; Meyers & Hill, 1983; Renard et al., 2000; Rutter, 1983; Scholle & Halley, 1985; Tada & Siever, 1989). Pressure‐solution occurs in the presence of a liquid phase through dissolution at grain contacts, diffusion of the solute matter, and precipitation of the latter within the pore spaces. The process is mainly driven by the stress acting at the grain‐to‐grain contact and does not require high ambient pressures and temperatures to be activated (Croizè et al., 2013; Rutter, 1983).

According to these observations, the textures found in the matrix of the slip zones associated with both normal faults and DGSDs are compatible with the activation of pressure‐solution processes occurring at very low temperatures and confining pressures (i.e., *T* < 15°, *P*_litho_ < 15 MPa). Cataclastic flow processes cause clast comminution by frictional sliding, grain crushing, and micro‐cracks growth. The subsequent ingression and percolation of fluids within the pore spaces (e.g., Lucca et al., 2019) among the calcite grains cause a very efficient pressure‐solution process resulting in grain indentation and stylolitic‐like grain boundaries, pore space reduction and precipitation of secondary calcite grains, and other phases within the fractures closest to the dissolution areas (Agosta et al., 2012; Bathurst, 1971; Carrio‐Schaffhauser et al., 1990; Renard & Ortoleva, 1997). The slip zones of San Benedetto‐Gioia dei Marsi and Roccapreturo normal faults underwent more pressure‐solution than the ones of Valle Force fault and of the DGSDs, probably because of the smaller average size of the grains (Renard et al., 2000; Rutter, 1983; Tada & Siever, 1989). However, other factors that influence the rate of pressure‐solution should be considered, such as the relative abundance of clay‐rich minerals, which were found in all the slip zones of the DGSDs, and the composition of the percolating fluids (Croizè et al., 2010; Renard et al., 2001; Tada & Siever, 1989).

The major slip surface of Valle Force fault shows ultra‐polished patches (where the hangingwall breccias are removed) and has a sharp contact with the underlying clasts of the slip zone (Figures 7f and 11a). The ultra‐polished surface patches could be the result of the formation of Y‐shears due to crack propagation and mechanical abrasion in a lithified fault rock (cataclasite). The mechanical abrasion produces a calcite‐rich nanopowder that is spread on the Y‐shears. The activation of pressure‐solution processes dissolving the finer calcite grains may result in the formation of an ultra‐polished surface (e.g., Mercuri et al., 2018; Tesei et al., 2017). Alternatively, ultra‐polished slip surfaces produced by extreme co‐seismic localization at larger crustal depths (De Paola et al., 2015; Demurtas et al., 2016; Fondriest et al., 2013; Pozzi et al., 2018) might be overprinted by low‐temperature pressure‐solution compaction during inter‐seismic periods and exhumation. This would suggest that, in fluid‐saturated systems, ultra‐polished slip surfaces are likely associated with textures produced by fluid‐driven and low temperature diffusive processes active on smooth surfaces during either seismic or aseismic slip. Recent experiments indicate that seismic slip at low effective stresses (i.e., <2 MPa and in the presence of pressurized pore fluids) is not able to induce crystal plasticity on carbonate gouges and to produce ultra‐polished slip surfaces (Rempe et al., 2020). Instead, carbonate fluid‐saturated gouges sheared at very low effective stresses likely deform by granular and, to a less extent, cataclastic flow during earthquakes (Rempe et al., 2020). These experimental results suggest that in the case of Alto di Cacchia DGSD that involves deposits that were not affected by significant burial (i.e., <400 m), the presence of polished slip surfaces might be the result of pressure‐solution processes.

## 6 Conclusions

In the Italian central Apennines, sharp, commonly karstified scarps displace carbonate rocks and accommodate either seismic ruptures or DGSDs. We analyzed the cases of Alto di Cacchia, Sant'Erasmo, Colle Cerese, and Mt. Serrone DGSDs, located in the footwall of large seismogenic normal faults, and the case of Valle Force, a <2 km long normal fault bordering a karst depression (Figures 1 and 2). In the footwall of the major scarp accommodating Alto di Cacchia DGSD, the fault/fracture network mainly consists of sub‐vertical open fissures: this attitude is consistent with the surficial formation (i.e., <500 m) of the fractures accommodating the DGSD (Figures 3 and 13). On the contrary, the fault/fracture networks associated with the other selected DGSDs consist of open fissures, joints, and shear fractures, but with a large scatter of the dip and dip angles. This scatter is indicative of a deeper formation depth (i.e., >1 km of depth) followed by the recent gravitational activity (Figures 4, 5, 6). In fact, a similar fault/fracture network is measured in Valle Force small normal fault (Figures 7 and 13). Therefore, we interpret most slip surfaces of the DGSDs in the central Apennines as the result of the exploitation of preexisting (tectonic‐related) faults/fractures located in the footwall of larger normal faults (Figures 13 and 14).

The maximum height values (up to 10 m) of the DGSD scarps are comparable to those of the main seismogenic normal faults (up to 10 km of length along‐strike) in the central Apennines. Therefore, well‐exposed high and sharp slip surfaces, also for large seismogenic faults, can be related to both tectonic faulting and gravity‐induced processes. However, structural, geomorphological and geophysical/seismological features, and information, such as the along‐strike length and lateral continuity of the scarp, the presence of double‐crested ridge or up‐hill facing scarps or gravitational trenches, the distribution of earthquakes, may allow us to relate the scarps to gravitational rather than to tectonic processes.

The slip zones of the large slip San Benedetto‐Gioia dei Marsi and Roccapreturo seismogenic normal faults have a cataclastic fabric and include a ∼0.5 cm thick continuous ultra‐cataclastic layer just beneath the slip surface (Figures 12a and 12d). On the other hand, the slip zones of Sant'Erasmo, Colle Cerese, and Mt. Serrone DGSDs have a proto‐cataclastic fabric (Figures 9a and 10a, c) and those of Alto di Cacchia DGSD and of Valle Force fault have a cataclastic fabric, but both lack of an ultra‐cataclastic layer right beneath the slip surface (Figures 8c and 11b). The well‐developed and thicker slip zones associated with the large normal faults can be explained by the larger amount of slip displacement accommodated by the latter compared to the DGSDs. However, other than these differences in the microstructural organization of the slip zone, there are no microstructural indicators that allow us to distinguish between DGSDs and normal faults.

The microstructures found in the fine matrix of the slip zone may yield information about the deformation mechanisms activated during DGSD. The fine matrix is composed of packed calcite micro‐ to nanograins with both straight and indented or even stylolitic‐like grain boundaries (Figures 8, 9, 10, 11). This fabric is interpreted as the result of cataclasis occurring by clast fragmentation and frictional sliding, and low temperature pressure‐solution processes (i.e., *T* < 15°, *P* < 15 MPa). The calcite micro‐grains forming the matrix of the large slip normal faults are more packed (e.g., well‐developed triple junctions, widespread indentation, and reduced number of pores) with respect to those found in the slip zones of DGSDs possibly because of the smaller average grain size that favors the process of pressure‐solution.

Our work stresses the structural convergence (i.e., formation of smooth to ultra‐polished slip surfaces) resulting from micro‐scale processes (i.e., cataclastic flow and pressure‐solution) active on both faults and DGSDs. Therefore, the characterization of the footwall fault/fracture network distribution, together with the interpretation of geomorphological features, are key inputs to relate the presence of sharp scarps to gravity‐ or tectonic‐driven processes.

## Data Availability

None of the data in our manuscript have been published or are under consideration elsewhere. The collected data set was uploaded and is available on http://researchdata.cab.unipd.it/id/eprint/423 (DOI: 10.25430/researchdata.cab.unipd.it.00000423).
